# Simultaneous NAD(P)H and FAD fluorescence lifetime microscopy of long UVA–induced metabolic stress in reconstructed human skin

**DOI:** 10.1038/s41598-021-00126-8

**Published:** 2021-11-12

**Authors:** Thi Phuong Lien Ung, Seongbin Lim, Xavier Solinas, Pierre Mahou, Anatole Chessel, Claire Marionnet, Thomas Bornschlögl, Emmanuel Beaurepaire, Françoise Bernerd, Ana-Maria Pena, Chiara Stringari

**Affiliations:** 1 Laboratory for Optics and Biosciences, Ecole polytechnique, CNRS, INSERM, IP Paris, 91128 Palaiseau Cedex, France; 2 L’Oréal Research and Innovation, 1 avenue Eugène Schueller BP 22, 93601 Aulnay-sous-Bois, France

**Keywords:** Optical physics, Nonlinear optics, Microscopy, Metabolism

## Abstract

Solar ultraviolet longwave UVA1 exposure of human skin has short-term consequences at cellular and molecular level, leading at long-term to photoaging. Following exposure, reactive oxygen species (ROS) are generated, inducing oxidative stress that might impair cellular metabolic activity. However, the dynamic of UVA1 impact on cellular metabolism remains unknown because of lacking adequate live imaging techniques. Here we assess the UVA1-induced metabolic stress response in reconstructed human skin with multicolor two-photon fluorescence lifetime microscopy (FLIM). Simultaneous imaging of nicotinamide adenine dinucleotide (NAD(P)H) and flavin adenine dinucleotide (FAD) by wavelength mixing allows quantifying cellular metabolism in function of NAD(P)^+^/NAD(P)H and FAD/FADH_2_ redox ratios. After UVA1 exposure, we observe an increase of fraction of bound NAD(P)H and decrease of fraction of bound FAD indicating a metabolic switch from glycolysis to oxidative phosphorylation or oxidative stress possibly correlated to ROS generation. NAD(P)H and FAD biomarkers have unique temporal dynamic and sensitivity to skin cell types and UVA1 dose. While the FAD biomarker is UVA1 dose-dependent in keratinocytes, the NAD(P)H biomarker shows no dose dependence in keratinocytes, but is directly affected after exposure in fibroblasts, thus reflecting different skin cells sensitivities to oxidative stress. Finally, we show that a sunscreen including a UVA1 filter prevents UVA1 metabolic stress response from occurring.

## Introduction

Solar ultraviolet (UV) rays constitute one of the most important environmental stresses to which human skin is constantly exposed. UV rays reaching the Earth surface are a combination of 5% UVB (290–320 nm) and 95% UVA (320–400 nm) including both shortwaves UVA (UVA2, 320–340 nm) and longwave UVA (UVA1, 340–400 nm). The Earth’s surface receives much less UVB than UVA because UVB are mostly absorbed by the ozone layer, the earth atmosphere, and the clouds. As a consequence, UVB exposure varies according to geographic and environmental parameters, whereas UVA exposure is always present, independently on meteorological conditions or on the outdoor vs indoor situation as UVA rays pass through windows and car glasses^1,2^. While UVB rays penetration is limited to the epidermis^3^, UVA rays penetrate deeper within skin dermis and can cause dermal damage with long-term consequences resulting in the appearance of photoaging clinical signs such as wrinkles, loss of skin elasticity, dyspigmentation and photocarcinogenesis^4^. Longwave UVA (UVA1, 340–400 nm) exposure has several important consequences both at cellular and molecular level as the photoexcited states of skin endogenous photosensitizers generate reactive oxygen species (ROS) (Figure S1)^5^. On reconstructed human skin, it was shown that UVA1 exposure generates ROS, which in turn induce oxidative damage, such as protein and lipid oxidation, membrane alterations and DNA damage, as well as several molecular alterations in the expression of an important number of genes related to innate immunity, oxidative stress, metabolism, inflammation or extracellular matrix remodeling^6^. Despite light-driven ROS formation is now widely accepted as contributor to skin photoaging and photocarcinogenesis^7–10^, the dynamic of the metabolic stress response in the skin, as well as its changes with UV exposure, remains largely unknown due to the lack of non-invasive techniques to monitor the energetic metabolism in live tissues.

Multiphoton microscopy of endogenous biomarkers has shown important potential for label-free and non-invasive monitoring of cellular metabolic processes in living tissues^11,12^. NAD(P)H and FAD are the most important metabolic cofactors of *redox* (reduction/oxidation) reactions in the cell and central regulators of mitochondrial ATP production and antioxidant defense^13,14^. The fluorescence lifetimes of NAD(P)H and FAD are exquisitely sensitive to enzyme binding during the cycling of the electron transport chain. The protein-bound NAD(P)H lifetime is significantly longer than the free NAD(P)H lifetime, due to self-quenching in the free state while the FAD lifetime is short and long in the protein-bound and free states, respectively^15–17^. Therefore, multiphoton FLIM imaging of the metabolic coenzymes NAD(P)H and FAD can provide functional information on cellular redox ratios (NAD(P)H/NAD(P)^+^ and FAD/FADH_2_) and on the complexity of several metabolic pathways (glycolysis, oxidative phosphorylation, oxidative stress, fatty acid oxidation and synthesis)^16,18–28^. While the nicotinamide adenine dinucleotide (NADH/NAD^+^) drives ATP production in the cytosol by glycolysis and in the mitochondria by oxidative phosphorylation, nicotinamide adenine dinucleotide phosphate (NADPH/NADP^+^) is mainly involved in the defense against reactive oxygen species^29,30^. It has been recently shown that the lifetime of NADH positively correlates with cellular Oxygen Consumption Rate (OCR)^31^ and fraction of bound/free NADH is proportional to NAD^+^ /NADH^16,20^. Since the emission spectra of NADH and NADPH species are identical, changes in NAD(P)H lifetimes pool may reflect changes in both species associated either with cellular energy production or antioxidant defense. Although quantitatively associating NAD(P)H and FAD lifetime changes to different metabolic cellular pathways remains challenging^32^ the two endogenous fluorophores are emerging as complementary biomarkers highlighting metabolic phenotypic heterogeneity^18,20,23,24^. Our group recently implemented simultaneous two-photon excitation of NAD(P)H and FAD by wavelength mixing to acquire FLIM data of the two biomarkers at the same time and perform multiparametric metabolic imaging in dynamic biological system^26^.

The goal of this study is to assess at different time scales the effects of UVA1 light exposure on the cellular metabolic activity of different cell types in reconstructed human skin using simultaneous fluorescence lifetime imaging of NAD(P)H and FAD. We used a reconstructed human skin in vitro model which includes a dermal equivalent with living fibroblasts and a fully differentiated epidermis. The 3D architecture of such skin model has been shown to be suitable for studying the effects of UV exposure in both epidermal keratinocytes and dermal fibroblasts^33^, especially regarding the oxidative stress response^34^. The skin metabolic state was assessed before and following UVA1 exposure with physiological doses (25 J/cm^2^ and 40 J/cm^2^), at 30 min and 2 h, in basal epidermal and superficial dermis layers. We present measurements of NAD(P)H and FAD fluorescence lifetimes changes after short-term exposure to UVA1 rays in keratinocytes and fibroblasts. We show that the metabolic response is biomarker, cell type and dose-dependent.

## Results

### Wavelength mixing, phasor and deep learning segmentation approaches for simultaneous NAD(P)H and FAD multiparametric metabolic FLIM analysis in reconstructed human skin.

**Figure 1:**
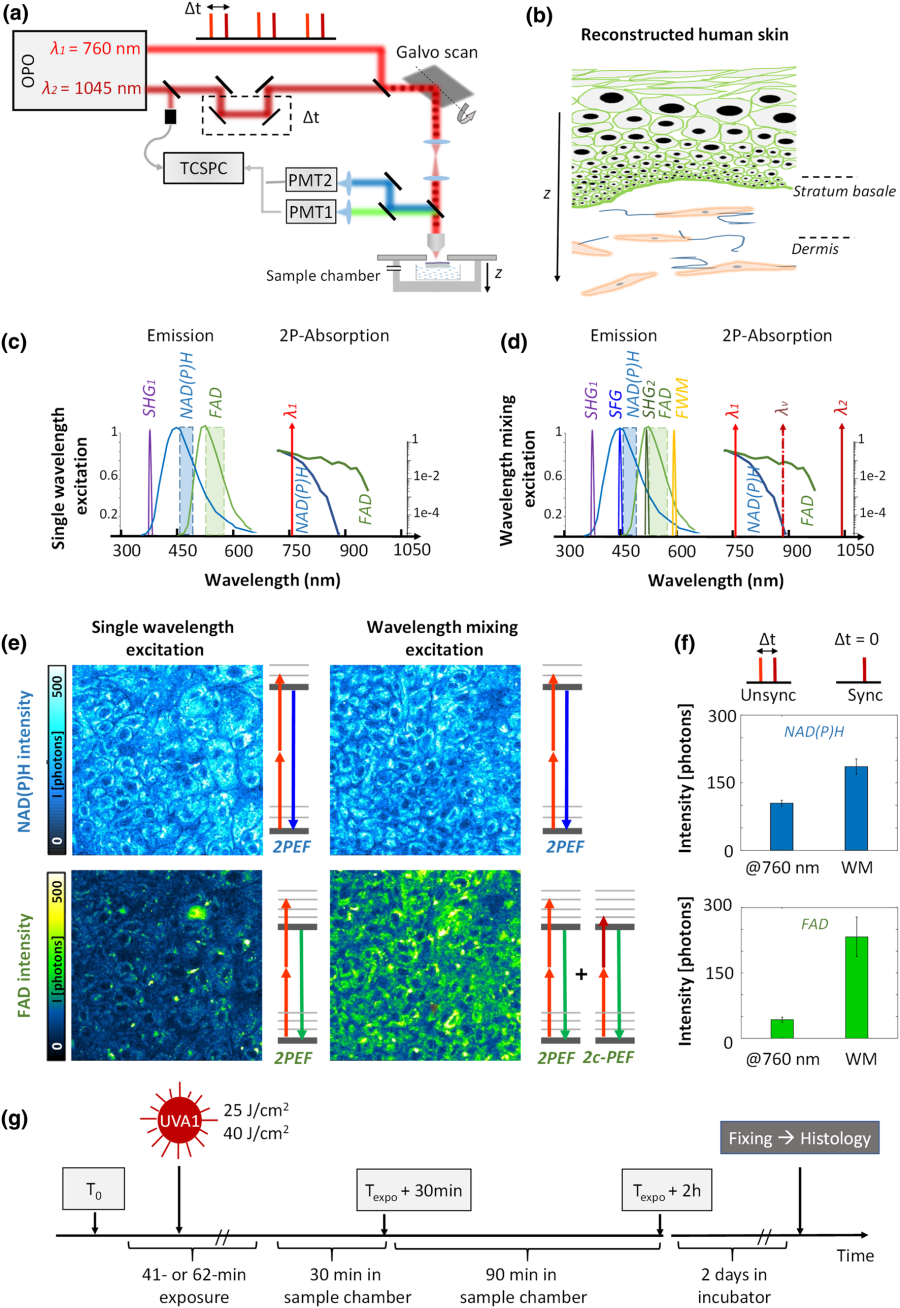
Multicolor two-photon excitation of NAD(P)H and FAD by wavelength mixing*.* (**a**) Schematic of two synchronized pulse trains from dual-output femtosecond laser used to generate one-color (2PEF) and two-color (2c-2PEF) nonlinear signals epi-detected in two separate spectral channels. The reconstructed human skin sample is kept in a temperature and CO_2_ controlled chamber. (**b**) Scheme of reconstructed human skin (T-Skin model) consisting of an epidermis (20–40 µm thickness) with keratinocytes at different differentiation states on top of a dermis layer (~ 1 mm thickness) containing fibroblasts embedded in a collagen fibrils substrate. Imaging is performed in the epidermis, within the *stratum basale* and in the upper dermis, at a depth of 20 µm below the dermal–epidermal junction as indicated by the dotted lines. (**c**–**e**) Single wavelength excitation at 760 nm enables both NAD(P)H and FAD imaging, but the resulting 2PEF FAD intensity is low due to the disparities in the biomarker’s concentration. Wavelength mixing (WM) excitation enables simultaneous and efficient 2P excitation of NAD(P)H and FAD by a virtual equivalent wavelength (dotted line) at 880 nm that optimally excites FAD biomarker independently of NAD(P)H. (**f**) Signal enhancement of the two fluorophores by wavelength mixing in the basal layer using unsynchronized and synchronized laser pulses. (**g**) Workflow of UVA1 exposure, FLIM imaging and histological analysis in reconstructed human skin.

To perform multiparametric metabolic imaging in reconstructed normal human skin (Fig. 1), we implemented simultaneous efficient excitation of the two intrinsic biomarkers NAD(P)H and FAD by wavelength mixing as previously demonstrated^26^. Two synchronous femtosecond laser beams at λ_1_ = 760 nm and λ_2_ = 1045 nm are temporally overlapped using a delay line (Fig. 1a and “Materials and methods” section). The spatial and temporal overlap of the two pulse trains gives rise to a third, virtual, two-photon excitation wavelength at λ_v_ = 2/(1/λ_1_ + 1/λ_2_) = 880 nm for two-color two-photon excitation (2c-2PEF) with one photon from each laser beam (Fig. 1d). 2c-2PEF increased FAD excitation efficiency (Fig. 1e) compared to a single wavelength 2PEF excitation at 760 nm without significantly changing NAD(P)H signal level (Fig. 1e) due to the low two-photon excitation of NADH at 880 nm. This strategy enables to control FAD and NAD(P)H signal levels independently while ensuring that all the fluorescence originates from the same diffraction-limited focal region. We optimized the power of the 1045 nm beam (see “Materials and methods”) to obtain an efficient simultaneous excitation of NAD(P)H and FAD and images with similar levels of photon counts (Fig. 1e, f).

Simultaneous NAD(P)H and FAD fluorescence lifetime microscopy was performed in live reconstructed human skin (T-Skin model reconstructed between 12 and 14 days of differentiation) within both the epidermal basal layer including undifferentiated keratinocytes and at 20 µm below the dermal–epidermal junction, in the superficial dermal layer constituted of fibroblasts embedded in a fibrillar collagen matrix (Fig. 1b). During imaging, the skin samples were kept in physiological conditions in a chamber with temperature and CO_2_ control (see “Materials and methods”). FLIM images were acquired before exposure (T_0_) and following UVA1 (25 J/cm^2^ or 40 J/cm^2^) or control sham-exposure at 30 min (T_expo_ + 30 min) and 2 h (T_expo_ + 2 h) (Fig. 1g and Materials and methods).

**Figure 2:**
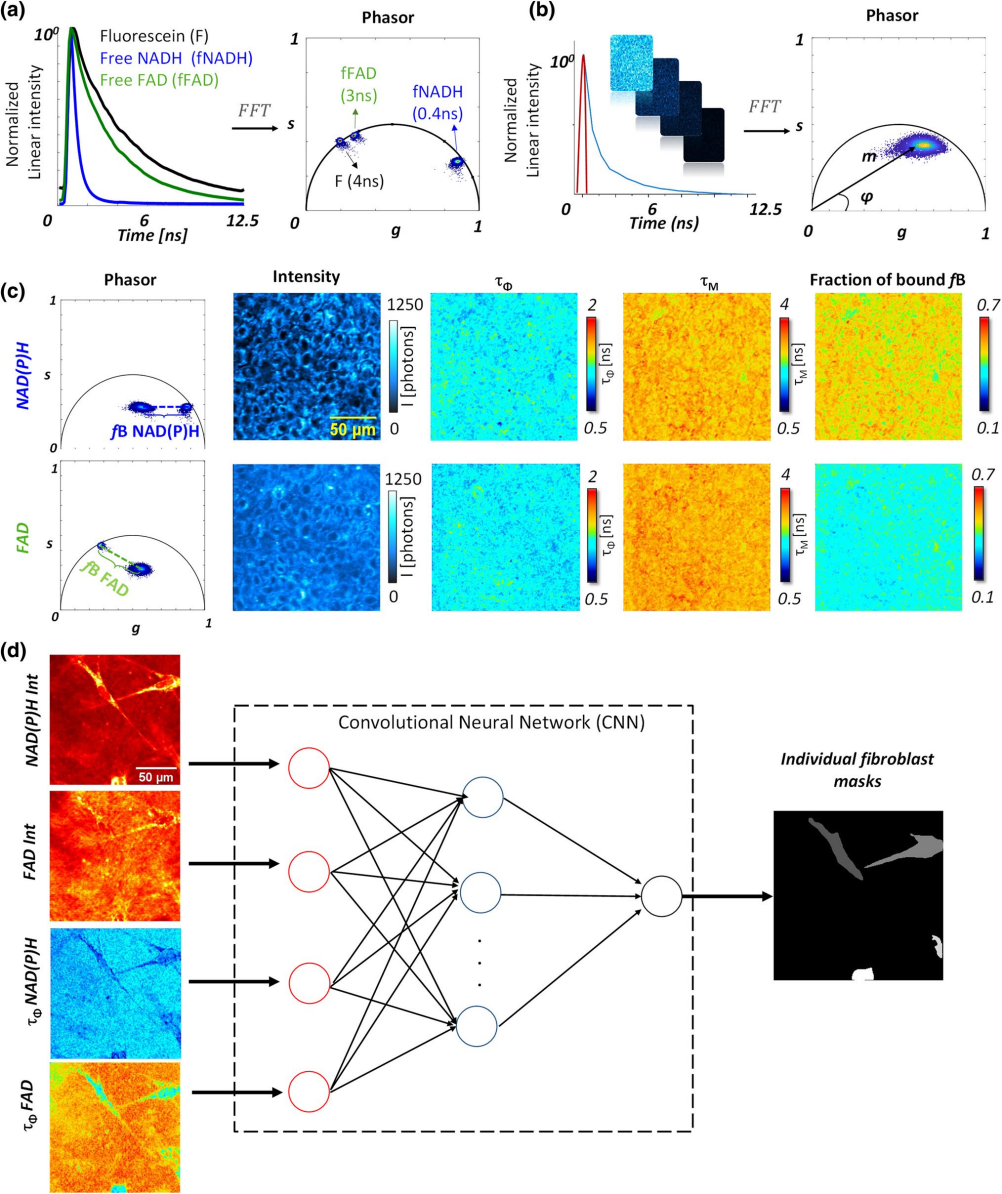
Image processing workflow for FLIM phasor analysis and single fibroblast segmentation with deep learning. (**a**) FFT based phasor analysis of FLIM images of fluorescein (F), free NAD(P)H (fNAD(P)H) and free FAD (fFAD) solutions with 4 ns, 0.4 ns and 3 ns lifetimes respectively. (**b**) Example of phasor analysis of the NAD(P)H FLIM image of keratinocytes within the *stratum basale* of T-Skin model. Every fluorescence decay (e.g. blue curve) of every pixel of the image is represented by a pixel in the phasor plot with g and s coordinates. The red curve indicates the instrumental response function. (**c**) Calculation of phase lifetime (τ_Φ_), modulation lifetime (τ_M_) and fraction of bound (*f*B) parameters of NAD(P)H and FAD based on g and s phasor FLIM parameters. The fractions of bound NAD(P)H and respectively bound FAD are calculated from the distances between the phasor plots of free NAD(P)H and respectively free FAD solutions to the mixed sample phasor plots of free / bound NAD(P)H and respectively free / bound FAD. (**d**) Workflow of single-fibroblast segmentation by deep learning with multi-modal images. Four inputs are given to the deep learning network: intensities and lifetimes of NAD(P)H and FAD. The output of the network provides a mask for every single cell.

Two channel Fluorescence Lifetime Microscopy was implemented as previously described^26^ with custom made time correlated single photon counting (TCSPC) electronics based on FPGA (see “Materials and methods”). We processed the lifetime decay in every pixel of the image with the phasor analysis based on fast Fourier transform (Fig. 2a–c and “Materials and methods”)^20^ and calculated the phasor plot as well as the phase lifetime (τ_Φ_) and the modulation lifetime (τ_M_) maps (Fig. 2b, c and supplementary Eqs. 5 and 6). To quantify the metabolic states of the cells, we mapped the relative concentration of free and bound metabolites in the tissue by first performing a calibration of free NAD(P)H and free FAD lifetimes in solution (Fig. 2a). For every pixel of the image, the fraction of bound NAD(P)H (*f*B NAD(P)H) and the fraction of bound FAD (*f*B FAD) were estimated by graphically measuring the distance of the experimental pixel in the phasor plot from the average location of free NAD(P)H and free FAD respectively (Fig. 2c and Figure S1c). FLIM calibration of the NAD(P)H metabolic trajectory under oxidative stress was performed in cell culture and we measured an increase in the fraction of bound NAD(P)H after application of hydrogen peroxide (Figure S1) showing that the fraction of bound NAD(P)H is proportional to the redox ratio of the cell NAD(P)^+^ /NAD(P)H.

**Figure 3:**
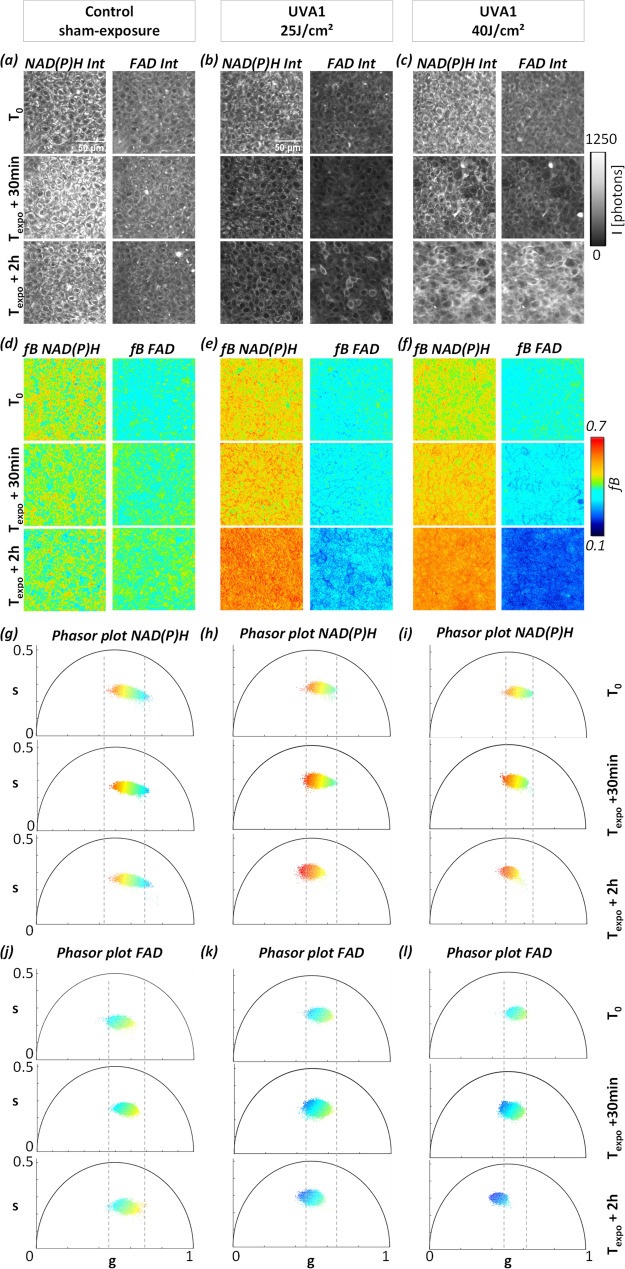
Temporal dynamic of UVA1 dose effects on NAD(P)H and FAD metabolic biomarkers in basal keratinocytes of reconstructed human skin. Representative images of (**a**–**c**) NAD(P)H and FAD fluorescence intensities and (**d**–**f**) *f*B fractions of bound NAD(P)H and FAD acquired before (T_0_), at 30 min (T_expo_ + 30 min) and 2 h (T_expo_ + 2 h) post sham-exposure (Control) or UVA1 exposure at 25 J/cm^2^ and 40 J/cm^2^. (**g**–**i**) Representative phasor plot scatters of NAD(P)H and (**j**–**l**) FAD fluorescence lifetime data of the same samples at T_0_, T_expo_ + 30 min and T_expo_ + 2 h. Every pixel of the phasor plot is color coded according to the fraction of bound metabolites with the same look up table as for images (**d**)–(**f**).

**Figure 4:**
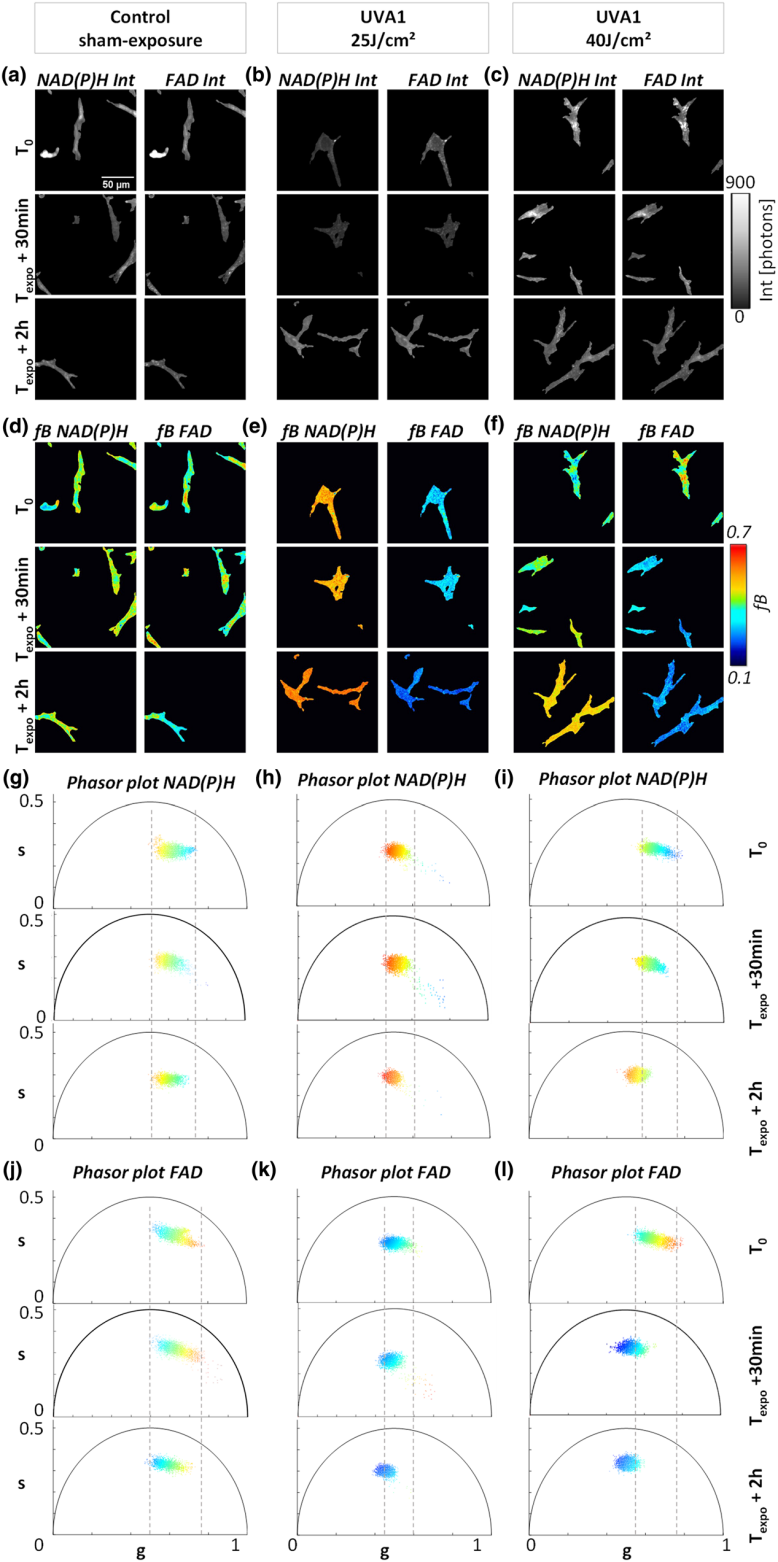
Temporal dynamic of UVA1 dose effects on NAD(P)H and FAD metabolic biomarkers in superficial dermis fibroblasts of reconstructed human skin. Representative images of (**a**–**c**) NAD(P)H and FAD fluorescence intensities and (**d**–**f**) *f*B fractions of bound NAD(P)H and FAD before (T_0_), at 30 min (T_expo_ + 30 min) and 2 h (T_expo_ + 2 h) post sham-exposure (Control) or UVA1 exposure at 25 J/cm^2^ and 40 J/cm^2^. (**g**–**i**) Representative phasor plot scatters of NAD(P)H and (**j**–**l**) FAD fluorescence lifetime data of the same samples at T_0_, T_expo_ + 30 min and T_expo_ + 2 h. Every pixel of the phasor plot is color coded according to the fraction of bound metabolites with the same look up table as for images (**d**)–(**f**).

Typical 2c-2PEF intensity images of the basal layer (Figs. 2c, 3a–c, 6a–c) show epidermal keratinocytes with a round shape and a typical diameter around 10 µm. NAD(P)H intensity images show higher signal in the cell cytoplasm coming from mitochondria and a dimmer nucleus and cellular membranes, while FAD intensity images present a more heterogeneous pattern with higher signals intensities in some regions. This type of FAD cellular distribution was also reported for an equivalent T-Skin model reconstructed at 18 days^35^, but the origin of these FAD high intensity signals remains unknown. Whether they originate from FAD binding to a specific protein complex^12^ and whether they are specific to reconstructed epidermis or also present in human skin in vivo remains to be investigated. The superficial dermis (typical images in Figs. 2d, 4a–c) shows elongated fibroblast cells embedded in a collagen fibrils substrate. Both NAD(P)H and FAD 2c-2PEF images show a homogenous signal intensity distribution within the cytoplasm, lacking the bright FAD signal intensities encountered in keratinocytes. In order to measure the metabolic activity in fibroblasts, an automated segmentation of single fibroblasts was implemented using a deep neuronal network^36^ (“Materials and methods”). The network was firstly trained on a set of manually segmented fibroblasts and the rest of the images database was processed by feeding the network with both intensity and τ_Φ_ lifetimes of NAD(P)H and FAD biomarkers to extract the masks of single cells (Fig. 2d).

We note that to properly collect the fluorescence of the two biomarkers, narrow emission filters had to be chosen to reject the coherent nonlinear signals of Second Harmonic Generation (SHG) created by each beam and sum frequency generation (SFG) and four wave mixing (FWM) arising from the mixing of the two beams (Fig. 1d). However, despite the band pass filter in front of the detectors (Fig. 1d), residual SFG and SHG signals from collagen fibrils were collected in the NAD(P)H and FAD channels respectively. This bleed-through resulted in an apparent shortening of the lifetime in the image background due to the presence of collagen fibrils, and also of the apparent lifetime of the biomarkers within the cells. For this reason, in the dermis, we only took into account the FLIM data that have been acquired exactly with the same laser power for both beams, as any variation of the power would introduce a bias in the estimated fluorescence lifetimes.

### NAD(P)H and FAD metabolic stress response to UVA1 exposure is specific to cell type and UVA1 dose

As evidenced by histological analysis, UVA1 exposure did not produce any visible changes in the epidermal or dermal morphology or fibroblast number in the reconstructed human skin samples analyzed at 30 min and 2 h post exposure (Figure S2a), while apoptosis of fibroblasts of the superficial dermis and thinning of the epidermis layer were observed at 2 days following UVA1 exposure (Figure S2b)^6^. Upon UVA1 exposure, we previously observed an increase in reactive oxygen species generation and lipid peroxidation in a dose dependent manner as well as a change in the expressions of genes associated to oxidative stress, inflammation, metabolism and many other functions^6^. We also demonstrated by sequential 2PEF FLIM imaging and bi-exponential decay analysis of NAD(P)H and FAD signals that total UVA (UVA2 + UVA1) light at 20 J/cm^2^ is able to induce, in T-Skin *strata basale* and *granulosum*, a decrease in the optical redox ratio NAD(P)H / (NAD(P)H + FAD) at 30 min and 2 h and an increase in the proportions of bound NAD(P)H at 2 h and free FAD as early as 30 min following exposure^35^. Hence, we sought to measure the metabolic stress response to the longwave region of UVA spectrum, i.e. UVA1, in a label-free way in reconstructed skin using two-color two-photon FLIM of the intrinsic metabolic biomarkers NAD(P)H and FAD and to determine the early changes occurring after exposure when ROS are generated, but visible morphological changes do not occur.

To approach the question whether UVA1 exposure has a specific effect on the metabolic state of basal epidermal keratinocytes and of fibroblasts in the superficial dermis, we studied reconstructed human skin samples exposed to different external stimuli: control (sham-exposure), UVA1 25 J/cm^2^, and UVA1 40 J/cm^2^ and we measured their metabolic fingerprint in both skin layers with 2c-2PEF FLIM of NAD(P)H and FAD at specific time points: before (T_0_) and following UVA exposure, at 30 min (T_expo_ + 30 min) and 2 h (T_expo_ + 2 h) (Figs. 3, 4).

In the epidermis, we chose to focus on undifferentiated keratinocytes in the basal layer because they have the same glycolytic metabolic phenotype independently from the differentiation state and thickness of the epidermis^26^.

Typical 2PEF intensity images of NAD(P)H and FAD biomarkers of keratinocytes (Fig. 3a–c) and fibroblasts (Fig. 4a–c) are represented under different UVA1 doses and at specific time points. For every condition, the maps of fraction of bound NAD(P)H (*f*B NAD(P)H) and fraction of bound FAD (*f*B FAD) (Figs. 3d–f, 4d–f) were calculated from the position of every pixel in the phasor plot (Figs. 3g–l, 4g–l) with respect to the free metabolites location (Fig. 2c). After UVA 1 exposure, an increase of the fraction of bound NAD(P)H and a decrease of the fraction of bound FAD were evidenced in both keratinocytes (Fig. 3d–f) and fibroblast (Fig. 4d–f) that correspond to an increase in both NAD(P)H and FAD lifetimes (Figs. 3g–l, 4g–l), whereas control sham-exposed samples did not show any change in lifetime and fraction of bound metabolites. Before exposure, the NAD(P)H and FAD phasor plots color coded by the *f*B parameter showed a broader distribution of phasors pixels with varying fractions of bound NAD(P)H and FAD indicating a subcellular heterogeneity of metabolic activity, also visible on the *f*B images. At 2 h post UVA1 exposure, the phasors pattern is more homogenous in terms of g, s and *f*B values and shifted towards longer lifetime values. A higher shift in FAD phasor plot is observed for the 40 J/cm^2^ UVA1 dose.

In order to quantify the metabolic response to UVA1, we calculated the average values of fractions of bound NAD(P)H (*f*B NAD(P)H) and bound FAD (*f*B FAD) and their corresponding phase lifetime (τ_Φ_) and modulation lifetime (τ_M_) in the basal layer by averaging the entire region of interest (ROI) that contains dozens of keratinocytes. Statistical analysis of NAD(P)H and FAD FLIM data was performed for a total of 814 ROIs for the basal layer (Figure S3, Figure S4, Figure S5, Figure S6, Figure S7 see “Materials and methods”). By averaging all the pixels within the ROIs, we are neglecting cellular heterogeneity that is present in the basal layer. Interestingly, we observed higher FAD intensity (Fig. 3b, c) and lower fraction of bound FAD (Fig. 3e, f) in some keratinocytes after UVA1 exposure demonstrating that the metabolic UVA1 response is heterogeneous and presents some variability among cells belonging to the same layer at the same depth. This heterogeneous metabolic UVA1 response is evidenced for all time-points with the 25 J/cm^2^ dose and at 30 min with the higher 40 J/cm^2^ dose. At 2 h post exposure with the higher UVA1 dose, all cells express the same homogenous metabolic response. On the other hand, after segmenting single fibroblasts, we performed single cell statistic in the dermis over 501 single fibroblast cells extracted from 407 ROIs (Figure S3, Figure S4, Figure S5, Figure S6, Figure S7).

**Figure 5:**
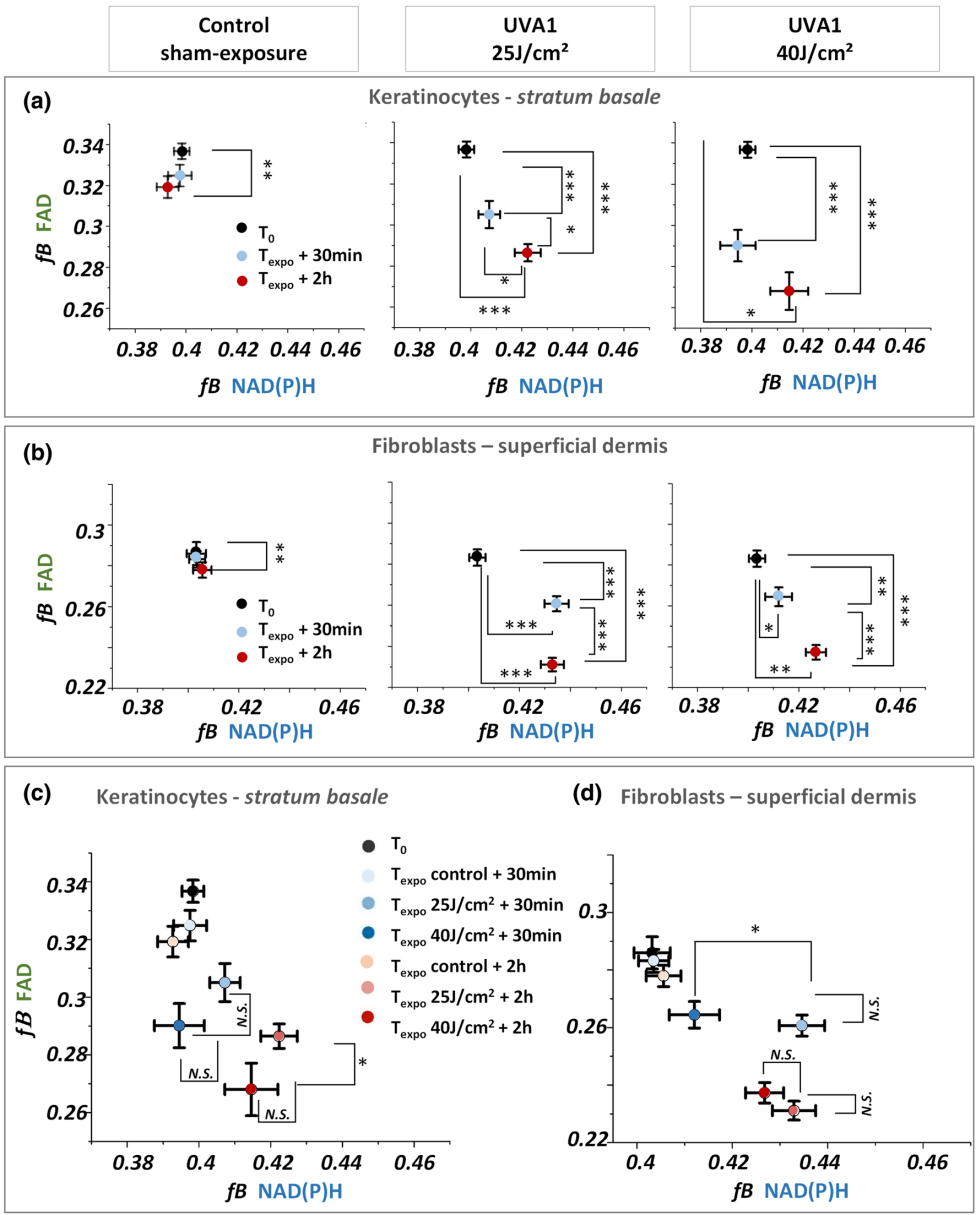
Temporal changes in the fractions of bound NAD(P)H (*f*B NAD(P)H) and bound FAD (*f*B FAD) in keratinocytes and fibroblasts of reconstructed human skin upon different UVA1 doses*.* (**a**, **b**) Fractions of bound NAD(P)H and bound FAD of basal keratinocytes (**a**) and superficial dermal fibroblasts (**b**) exposed to different UVA1 doses or control-sham exposure. (**c**)–(**d**) Same quantification data as in (**a**) and (**b**), regrouped to highlight the sensitivity to UVA1 doses of NAD(P)H and FAD biomarkers in the basal (**c**) and dermal layers (**d**) of reconstructed human skin. Data are presented as mean ± s.e.m. N.S. indicates distributions that are not statistically different and * indicates distributions that are statistically different (*p < 0.05, **p < 0.01 and ***p < 0.001).

To decipher the dynamic changes of the two biomarkers, we compared the evolution of fractions of bound NAD(P)H and FAD in function of both post exposure time and UVA1 dose (25 and 40 J/cm^2^). Two hours after exposure, we observed an increase of the fraction of bound NAD(P)H in both basal keratinocytes and superficial dermis fibroblasts with the two doses (Fig. 5a, b and Figure S4) that corresponds to an increase of the τ_Φ_ lifetimes (Figure S5). The increase in the fraction of bound NAD(P)H reflects an increase of the cellular redox NAD(P)^+^ /NAD(P)H ratio upon UVA1 stress response indicating either an increase in oxidative phosphorylation or in oxidative stress (Figure S1)^20,37^. Interestingly, we observed unique temporal dynamic in the metabolic response of NAD(P)H biomarker that depends on the cell type: while in fibroblasts of the superficial dermis we observed an increase of the fraction of bound NAD(P)H already at 30 min after exposure, the keratinocytes in *stratum basale* do not show any metabolic response at this earlier time point. The different temporal dynamic of NAD(P)H metabolic response might indicate a higher sensitivity and response of fibroblasts to oxidative stress as shown in earlier studies^34,38^. We observed no dependence of bound NAD(P)H response in keratinocytes on the UVA1 doses, probably suggesting that the NAD(P)H biomarker’s response at 2 h post UVA1 exposure is already at its maximum with the 25 J/cm^2^ dose, while in fibroblasts we observed a different response for 25 and 40 J/cm^2^ UVA1 doses at 30 min after exposure (Fig. 5c, d and Figure S8). The increase in bound NAD(P)H in fibroblasts was higher at 30 min after exposure with the lower dose, attaining the same level of response as at 2 h with both UVA1 doses.

Next, we examined the metabolic response of the FAD biomarker and observed a different temporal dynamic and different UVA1 dose-dependence with respect to the NAD(P)H biomarker. Both keratinocytes and fibroblasts, exhibited a gradual decrease in the fraction of bound FAD and increase of τ_Φ_ lifetimes, starting 30 min after exposure with both 25 and 40 J/cm^2^ UVA1 doses (Fig. 5a, b, Figure S4). Interestingly in keratinocytes at 2 h following exposure, a higher decrease in the fraction of bound FAD was evidenced with 40 J/cm^2^ than with 25 J/cm^2^ (Fig. 5c) that was not observed at 30 min after exposure. In fibroblasts, the temporal changes of FAD biomarker are not dose-dependent (Fig. 5d) but they are time-dependent, which suggests that their metabolic response is already at its maximum with the 25 J/cm^2^ UVA1 dose. These results reveal a time-dependent change of FAD biomarker in both keratinocytes and fibroblasts, a sensitivity to UVA1 dose 2 h after exposure in keratinocytes and no UVA1 dose effect in fibroblasts.

### Sunscreen with UVA1 filtration prevents NAD(P)H and FAD metabolic stress responses to UVA1

**Figure 6:**
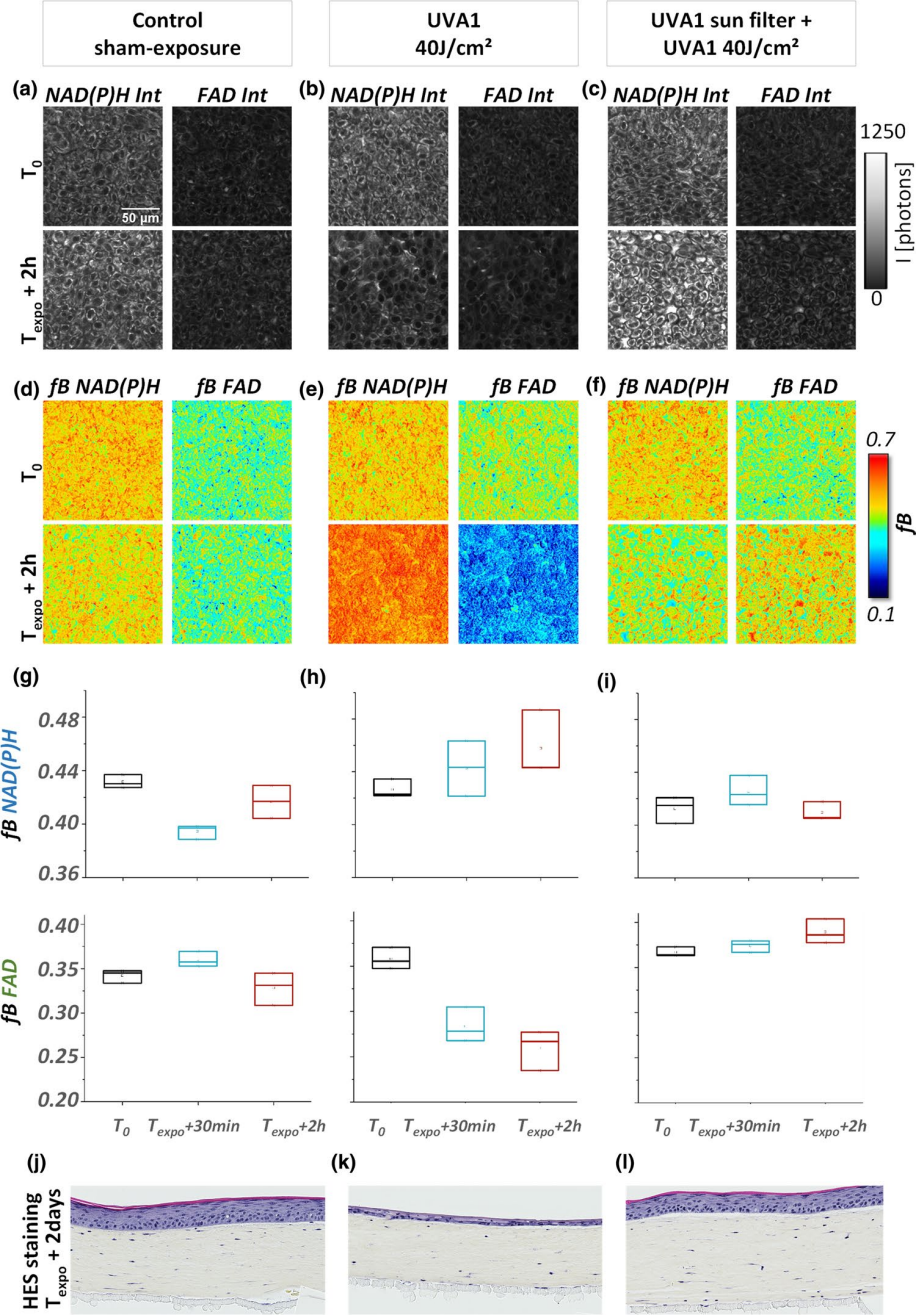
Effect of a long UVA filter on NAD(P)H and FAD metabolic stress response to UVA1. (**a**–**c**) Example of NAD(P)H and FAD fluorescence intensities and (**d**–**f**) fractions of bound NAD(P)H and FAD before (T_0_) and 2 h (T_expo_ + 2 h) post sham-exposure (Control), UVA1 40 J/cm^2^ exposure in the presence and absence of a long UVA sun filter. (**g**–**i**) Quantification of fractions of bound NAD(P)H and FAD of the same samples at three experimental time points (T_0_, T_expo_ + 30 min and T_expo_ + 2 h). Data are presented as boxplots. (**j**–**l**) Histological HES (hematoxylin eosin saffron) analysis of the same reconstructed human skin samples imaged by FLIM, replaced in culture and fixed at 2 days post exposure.

Next, we sought to determine the effect of a broad spectrum sunscreen formulation including the long UVA filter MCE on the metabolic stress response of the skin and performed 2c-2PEF FLIM of NAD(P)H and FAD biomarkers in reconstructed human skin exposed to UVA1 in the presence and absence of the long UVA sunscreen. Figure 6a–c shows representative 2PEF NAD(P)H and FAD intensity images of keratinocytes under three conditions: control (sham-exposure), UVA1 40 J/cm^2^, and sunscreen + UVA1 40 J/cm^2^. All measurements were performed at three time points: before UVA exposure (T_0_), 30 min after exposure (T_expo_ + 30 min) and 2 h after exposure (T_expo_ + 2 h). The corresponding maps of fractions of bound *f*B NAD(P)H and *f*B FAD under the three conditions are presented in Fig. 6d–f. Statistical analysis of *f*B NAD(P)H and *f*B FAD was performed over 27 ROIs, with 3 ROIs for each time point (Fig. 6g–i).

While *f*B NAD(P)H increased and *f*B FAD decreased progressively following long UVA1 40 J/cm^2^ exposure (Fig. 6e, h), we did not observe any changes in the fractions of bound NAD(P)H and FAD in the skin samples protected by the long UVA filter (Fig. 6f, i) nor in the control-sham exposed samples (Fig. 6d, g). This result shows that NAD(P)H and FAD metabolic stress responses to UVA1 exposure is prevented by using the sunscreen (Fig. 6f, i). The absence of UVA-1 induced early metabolic stress response was correlated with a complete protection of the structure of reconstructed skin as assessed by histology at 2 days following exposure (Fig. 6j–l). Compared to control-sham exposed samples (Fig. 6j), the UVA1 exposed samples (Fig. 6k) show a clear epidermis atrophy and fibroblasts disappearance within the superficial dermis. On the contrary, we observed no morphological alterations in the skin samples exposed to 40 J/cm^2^ UVA1 in the presence of the sunscreen (Fig. 6l), characterized by a skin morphology close to that of the control-sham exposed samples (Fig. 6j).

## Discussion and conclusions

In this work, we implemented a label-free, non-invasive multiparametric metabolic imaging based on simultaneous two-photon excitation of NAD(P)H and FAD biomarkers and fluorescence lifetime microscopy^26^ to study long UVA-induced metabolic stress in reconstructed human skin. We first optimized NAD(P)H and FAD excitation efficiencies by enhancing FAD signal level independently of NAD(P)H through two-color two-photon excitation (Fig. 1) by wavelength mixing^39^ obtaining comparable fluorescence intensities with a good signal-to-noise ratio in the two channels, in a one-shot measurement and without motion artifacts (Fig. 2c). We applied this approach to the simultaneous measurement of NAD(P)H and FAD lifetimes in keratinocytes in the epidermis basal layer and in fibroblasts in the superficial dermis layer of reconstructed human skin exposed to different doses of UVA1 light or control-sham exposure (Fig. 1b). To quantify the cellular metabolic states associated to changes in oxidative phosphorylation, glycolysis and oxidative stress rates, we performed a phasor analysis of the FLIM data^20^ and mapped the phase (τ_Φ_) and modulation (τ_M_) lifetimes of both NAD(P)H and FAD biomarkers and the fractions of bound metabolites (Fig. 2c and Figure S1). We then implemented an automated fibroblast segmentation using a deep learning approach (Fig. 2d) to perform single cell statistical analysis in the dermis layer.

We investigated the temporal dynamic of NAD(P)H and FAD biomarkers response (before, 30 min and 2 h after exposure) and the biomarkers sensitivity to UVA1 dose (25 and 40 J/cm^2^). After performing non-invasive live imaging, we kept the imaged in vitro reconstructed human skin samples in physiological conditions for 2 days and assessed the morphological and fibroblast number changes by histology HES staining analysis (see “Materials and methods”). While no morphological or cellular alterations appear in the skin at the early 30 min and 2 h post exposure time points (Figure S2a), after 2 days a thinning of the epidermis layer and fibroblast apoptosis in the superficial layer of the dermis occur (Figure S2b). We observed a progressive increase in both NAD(P)H and FAD lifetimes at 30 min and 2 h after UVA1 exposure (Figs. 3, 4) demonstrating the sensitivity of these endogenous biomarkers to long UVA-induced stress at very early time points when no visible changes are detected by histology HES staining (Figure S2). The increase of fraction of bound NAD(P)H and the decrease of fraction of bound FAD in both keratinocytes (Fig. 3) and fibroblasts (Fig. 4) are indicating an increase of NAD(P)^+^/NAD(P)H and FAD/FADH_2_ redox ratios, suggesting an increase of either oxidative phosphorylation (associated to the NADH pool) or oxidative stress (associated to the NADPH pool) as measured in the metabolic trajectory with an oxidative treatment (Figure S1). Previous work on the same reconstructed skin samples showed that total UVA (UVA2 + UVA1) light at 20 J/cm^2^ is also able to induce changes in both NAD(P)H and FAD lifetimes parameters, i.e. a decrease in the optical redox ratio NADH/(NAD(P)H + FAD) at 30 min and 2 h, an increase in the fraction of bound NAD(P)H at 2 h and free FAD as early as 30 min, in both *strata basale* and *granulosum*^35^. Other studies evidenced an increase of optical redox ratio FAD/(FAD + NADH)^40^ in ex vivo human skin samples exposed to total UVA light and a different change in NADH lifetime induced by UVA in ovary^41^ and UVB in fibroblasts^42^ cell cultures.

In this study, we demonstrated that NAD(P)H and FAD biomarkers have unique temporal dynamic after UVA1 exposure and specific sensitivity to skin cell types and UVA1 dose, therefore providing complementary information on the metabolic stress response of reconstructed human skin (Fig. 5). NAD(P)H biomarker showed an early change at 30 min after exposure in dermal fibroblasts, while a significant change in epidermal keratinocytes was only observed at 2 h after exposure (Fig. 5 and Figure S3, Figure S4, Figure S5, Figure S6, Figure S7, Figure S8). That could be the consequence of the different sensitivity and response of the two cells types to oxidative stress^34,38^, both cell types having differences in basal anti-oxidant defense equipment^43,44^. FAD biomarker showed an early change at 30 min after exposure in both cell types demonstrating its higher sensitivity to UVA1-induced metabolic stress in keratinocytes with respect to NAD(P)H biomarker. Interestingly, we observed that FAD lifetime is sensitive to the UVA1 dose in the epidermis (Fig. 5, Figure S7, Figure S8) when different amounts of ROS are generated by different UVA1 doses^6^, possibly because FAD is a photosensitizer^5^. Finally, we showed that NAD(P)H and FAD metabolic changes associated with UVA1 exposure were prevented in reconstructed human skin when using an appropriate broad spectrum sunscreen formulation containing a UVA1 filter allowing to have a complete UV absorption profile (Fig. 6).

In conclusion, in this study we performed the first extensive characterization of metabolic stress response in the epidermis and dermis of in vitro reconstructed human skin after control-sham and long UVA1 exposures using label free multicolor multiphoton fluorescence lifetime imaging of NAD(P)H and FAD biomarkers. We note that the investigation of the NAD(P)H and FAD biomarkers sensitivity dependence on keratinocytes differentiation state and baseline metabolic state is beyond the scope of this work. We also note that we characterized UVA1-induced metabolic stress in a skin model without melanocytes, the cells producing melanin. Therefore, in the presence of melanin, it may be necessary to further isolate signals from NAD(P)H and FAD based on their spectrum and/or lifetime^45–47^. Moreover the presence of different types of melanin could modulate the UVA1-induced metabolic stress as pheomelanin is a photosensitizer while eumelanin is photoprotective^5^.

Overall, our results demonstrate the sensitivity of this combined fluorescence lifetime signature to cell type and UVA1 dose, and the potential of this method to characterize in a label-free manner metabolic spatio-temporal changes in tissue photobiology. Multiphoton FLIM metabolic activity imaging also holds promising perspectives for in vivo non-invasive monitoring of human skin metabolic stress response to different UV light exposures and efficacy evaluation of anti-oxidant molecules and sunscreens.

## Materials and methods

### Two-color two-photon excitation fluorescence lifetime imaging (2c-2PEF-FLIM) of endogenous fluorophores in living tissues by wavelength mixing

Imaging was performed on a lab-built laser scanning two-photon microscope equipped with wavelength mixing and FLIM capabilities. A simplified schematic of microscope is presented in Fig. 1a. The excitation was performed by a dual-output infrared femtosecond laser source (Insight X3, Spectra-Physics, Santa Clara, CA, USA) with a tunable output from 680 to 1300 nm (120 fs pulses, 80 MHz) and a fixed output at 1045 nm (200 fs pulses). We used a dichroic mirror (Semrock, Rochester, NY, USA) to combine the two beams. Beams powers were controlled with two independent motorized wave plates and polarizers (Semrock, Rochester, NY, USA). To perform wavelength mixing, the two beams were spatially overlapped at the sample plane using independent telescopes and mirrors^39^, and pulses were temporally overlapped using a delay line placed in the 1045 nm optical pathway. The synchronization of two pulse trains provided an additional two-photon equivalent (‘virtual’) wavelength at λ_v_ = 2(1/λ_1_ + 1/λ_2_) that corresponds to 880 nm. The NAD(P)H fluorescence was excited at 760 nm with a typical power of 1 mW, while the virtual wavelength at 880 nm excited FAD fluorescence (Fig. 1d) independently of NAD(P)H. The power of the 1045 nm laser beam was set to 30 mW. A water immersion objective (25X, 1.05NA, XLPLN25XWMP, Olympus, Tokyo, Japan) was used to focus the beam on the sample and collect fluorescence signals. Fluorescence signals are epi-detected in two different spectral channels (Hamamatsu H7422-40 GaAsP detectors). Band-pass filters were used in front of these detectors to collect NAD(P)H (Semrock FF01–466/40) and FAD (Semrock FF01–550/49) respectively.

A fast discriminator (Hamamatsu C9744 photon counting unit) was used to detect the pulses of the detectors. FLIM data were acquired simultaneously in these two channels with a lab-designed counting electronics enabling time-correlated single photon counting. The laser pulse trigger of the fixed output was taken from the Insight X3. The arrival time between the photon and the laser pulse was measured using a time to digital converter (TDC), implemented in a Xilinx Spartan-6 FPGA on the electronic card. The TDC uses for each channel, 2 parallelized deserializer, respectively clocked at the X12 laser frequency and X12 phase shifted by 180° with one Digital Clock Managers of the FPGA. With a laser period of 12.48 ns, the histogram of these times measure was built with 2 × 12 = 24 temporal bins of 12.48 ns/24 = 0.52 ns width. Calibration of the FLIM system was performed by measuring the lifetime of the SHG (0 ns) and fluorescein at pH 9 (single exponential of 4 ns) (see Fig. 2a). The laser was scanned by galvanometric mirrors (VM500S, GSI Lumonics, Bedford, MA, USA), then the acquisition was synchronized using lab-written LabVIEW software and a multichannel I/O board (PCI-6115, National Instruments, Austin, TX, USA). We typically acquired between 400 and 1000 photons in FLIM images of 320 × 320 pixels with a pixel dwell time of 80 µs/pixel for a total acquisition time of 98 s (4 images with 20 µs/pixel are accumulated).

We imaged a total of 46 samples of reconstructed human skin: 21 for control (sham-exposure), 12 for UVA1 25 J/cm^2^, 10 for UVA1 40 J/cm^2^ and 2 for 40 J/cm^2^ with sunscreen. Every skin sample was imaged at three different time points: before exposure (T_0_) and at 30 min (T_expo_ + 30 min) and 2 h (T_expo_ + 2 h) after UVA1 exposure. For every skin sample and time point, we acquired 3 ROIs in the basal layer and 3 ROIs in the dermis layer (see the total number of ROIs per condition in supplementary table 1 and supplementary table 2). We performed imaging in reconstructed human skin between 12 and 14 days of differentiation, with a varying 20–40 µm epidermal thickness. As imaging is performed in the basal epidermal layer and in the dermis at 20 µm below the dermal–epidermal junction, the imaging depth varies depending on the epidermal thickness and differentiation state of the reconstructed human skin. Given that UVA1 rays penetrate deeper within human skin dermis and that this skin model has a thinner epidermal compared to human skin, we assume that the small difference in thickness at 12 and 14 days of differentiation does not significantly affects the UVA1 exposure to the imaged layers.

### Data analysis and processing of FLIM images

All intensity data were analyzed and treated with ImageJ (NIH, Bethesda, MD, USA). FLIM phasor analysis was performed with a Matlab (Mathworks, Natick, MA, USA) custom written software. Every pixel of the FLIM image was transformed in one pixel in the phasor plot as previously described^20,48^ and reported in detail in the supplementary information. The coordinates *g* and *s* in the phasor plot were calculated from the fluorescence intensity decay of each pixel of the image (Fig. 2b) by using the transformations defined in the supplementary material (Eqs. 1 and 2). We applied a median filter on the *g* and *s* images to reduce the variance of the phasor location without decreasing the spatial resolution of the image^49^. For every pixel of the image, we calculated the values of phase τ_Φ_ and modulation τ_M_ lifetimes and fractions of bound NAD(P)H (*f*B NAD(P)H) and bound FAD (*f*B FAD) (Eq. 5, 6, 7 and 8 respectively in supplementary information). The calculation of fraction of bound NAD(P)H and fraction of bound FAD was performed by measuring the distance between the experimental point and the phasor location of the free coenzyme, neglecting differences in the quantum yield between the free and enzyme-bound metabolites. We represented the FLIM data with the τ_Φ_, τ_M_, *f*B maps of NAD(P)H and FAD (Fig. 2c).

### Single-fibroblast segmentation with deep learning

We used a deep convolutional neural network (CNN) to segment the fibroblasts within the fibrillary collagen matrix of reconstructed human skin dermis. The main advantage of CNN was that it could easily cope with multimodal images, not to mention its performance. Since we could benefit from multimodal images through FLIM analysis, it was important to make use of their correlation. Each set of inputs was constructed by stacking NAD(P)H and FAD intensity images along with their phase lifetime images. We slightly modified a popular deep CNN, namely U-Net^36^ for segmenting foreground, and afterwards applied a simple labelling technique to separate instances of fibroblasts.

A subset of images with manually segmented mask labels was used for training the network. For the preprocessing, after calibration, we clipped pixel values above 0.99 quantile for each mode to get rid of big outliers, and normalized them with their maximum values so that pixel values from all modalities ranged between 0.0 and 1.0. In addition, we applied a set of simple gamma and log contrast augmentation during the training of the network, because there were big variations of contrast between foreground and background over samples, especially by date.

We defined the foreground segmentation mask by setting a threshold at 0.5 on the prediction, which ranges from 0.0 to 1.0 due to a softmax function at the end of the network. After, each pixel was labelled so that a set of connected pixels had the same label based on their 8 neighbouring pixels; left, right, up, down and 4 diagonal pixels. Even though it was a simple method, it yielded great results because fibroblasts were very sparse, they usually occupied a large portion within a viewpoint, and they rarely overlapped. Following this, too small labels whose area was less than a threshold got removed, because most of them were false positive. The area threshold was deduced from the manual annotation. Finally, the resulted segmentation masks were used to measure characteristics of fibroblasts.

### In vitro reconstructed human skin – T-Skin model

Reconstructed human skin samples (T-Skin model) at 11 days of differentiation were purchased from Episkin (Lyon, France). T-Skin is a full thickness skin model containing both living dermis and epidermis with a well-structured *stratum corneum*. The dermal layer contains normal human fibroblasts embedded in a collagen fibrills substrate supporting a fully differentiated epidermal keratinocyte layers: the basal layer, located right above the dermal substrate, contains proliferating undifferentiated cells, while the uppermost layer, the *stratum corneum*, contains fully differentiated keratinocytes, i.e. corneocytes. This model is histologically very similar to the in vivo human skin and displays correct epidermal differentiation process. The T-Skin samples, reconstructed at day 12 and 14 days, were placed between two glass coverslips with a few µl of culture medium (Episkin Assay Medium, Episkin, Lyon, France) and imaged immediately after preparation. For live multiphoton FLIM imaging, the skin was placed onto a homemade plastic grid holder (see Fig. 1), inside an adhesive silicone seal with a glass coverslip on top and the entire support was positioned inside a Petri dish, containing a non-fluorescent assay medium (Episkin Assay Medium, Episkin, Lyon, France). The entire device has been placed inside an incubation chamber at 37 °C and 5% CO_2_ (Okolab, Pozzuoli, Italy).

### UVA1 exposure

Solar UV exposure can be reproduced in the laboratory using a xenon-arc solar simulator (Oriel, Les Ulis, France) and appropriate filters, enabling UVA1 (340–400 nm) emission^6^. In this study, the skin samples were exposed during 42 min corresponding to an UVA1 dose of 25 J/cm^2^ and 65 min corresponding to an UVA1 dose of 40 J/cm^2^. During UVA1 exposure the assay culture medium was replaced by phosphate-buffered saline (PBS) solution and the glass coverslip cover was removed. The control-sham exposed samples were maintained in the same environmental conditions as the UVA1-exposed samples, except that the shutter of the solar simulator was closed during the duration of the control-sham exposure. After imaging, the samples were replaced in culture for 2 days and processed for histology HES (hematoxylin eosin saffron) staining analysis (Fig. 1g).

### Sunscreen and application protocol

The sunscreen contained the following UV filters: 5% Octocrylene, 0.8% Avobenzone, 1.1% Tinosorb S and 1.5% of the new UVA1 filter Methoxypropylamino Cyclohexenylidene Ethoxyethylcyanoacetate (MCE)^50^. This composition enabled the filtration of UVB, UVA2 and UVA1 wavelengths up to 400 nm, in a well-balanced manner^51^.

During UVA1 exposure in the presence of sunscreen product, the reconstructed skin samples were covered by a 5 × 5 cm^2^ PMMA plate (HD6 type, Helioscreen, Creil, France) on which 1.2 mg/cm^2^ of sunscreen was applied 15 min prior to UVA1 exposure.

### Solution preparation

A solution of 250 μM free NADH (Sigma Aldrich n.N8129, St. Louis, MO, USA) was prepared in 100 mM Mops buffer at pH 7. The free FAD solution (Sigma Aldrich n.F6625, St. Louis, MO, USA) was diluted at 2 mg/ml in water. Fluorescein solution was prepared in pure water with pH 9.

## Supplementary Information

Supplementary Information.
