# Supplementary material for: Simultaneous NAD(P)H and FAD fluorescence lifetime microscopy of long UVA–induced metabolic stress in reconstructed human skin
Source: Sci Rep. 2021 Nov 12;11:22171. doi: 10.1038/s41598-021-00126-8 (PMC8589997; doi:10.1038/s41598-021-00126-8)
Supplement: Supplementary file 1 — Supplementary Information. [file 41598_2021_126_MOESM1_ESM.docx]

**SUPPLEMENTARY INFORMATION**

The fluorescence lifetime intensity decays in every pixel of the image are transformed in the phasor plot through a Fourier transform. The components *g* (x‐coordinate) and *s* (y‐coordinate) are given by the following expressions:

$g_{i,j}\left( \omega\right)=\frac{\int_{0}^{\infty} I_{i,j}\left( t \right)\cos\left( \omega t \right)dt}{\int_{0}^{\infty} I_{i,j}\left( t \right)dt}$ (1)

$s_{i,j}\left( \omega\right)=\frac{\int_{0}^{\infty} I_{i,j}\left( t \right)\sin\left( \omega t \right)dt}{\int_{0}^{\infty} I_{i,j}\left( t \right)dt}$ (2)

where the indices *i* and *j* identify a pixel of the image and *ω* the frequency (𝜔=2𝜋𝑓), where *f* is the laser repetition rate, *i.e.* 80 MHz in our experiment. All phasor plots are calculated at 80 MHz, *i.e.* the first harmonic of the laser repetition rate.

The phasor transformations of FLIM data in the frequency domain are:

| 𝑔_𝑖,_(𝜔)=𝑚_𝑖_,_𝑗_cos𝜑_𝑖,j_ | (3) |
| --- | --- |
| 𝑠_𝑖_,(𝜔)=𝑚_𝑖_,_𝑗_sin𝜑_𝑖,j_ | (4) |

where $m_{i,j}$and $\varphi_{i,j}$ are the modulation and the phase of the emission with respect to the excitation. Estimations of the lifetime in terms of the phase and modulation can be performed in each pixel by the following formulas:

$\tau_{\varphi}=\frac{1}{\omega}tan\left( \varphi\right)$ (5)

$\tau_{m}=\frac{1}{\omega}\sqrt{\left( \frac{1}{m^{2}}-1 \right)}$ (6)

In the case of a single exponential decay, the two lifetimes obtained by the phase and by the modulation with equations (5) and (6) are equal, while for a multi-exponential lifetime system the apparent lifetimes are different.

To quantify the redox ratio NAD(P)H/NAD(P)^+^ and FADH/FAD, we calculated the fraction of bound NAD(P)H and fraction of bound FAD by measuring the distance of the experimental point in the phasor plot from the location of free NAD(P)H and free FAD respectively (Figure 2a and 2c) by using the following formulas:

| $fB\_NAD(P)H=\sqrt{\left( g_{exp\_NAD(P)H}-g_{fNADH} \right)^{2}+\left( s_{exp\_NAD(P)H}-s_{fNADH} \right)^{2}}$ | (7) |
| --- | --- |
| $fB\_FAD=\sqrt{\left( g_{exp\_FAD}-g_{fFAD} \right)^{2}+\left( s_{exp\_FAD}-s_{fFAD} \right)^{2}}$ | (8) |

We note that when calculating the fraction of bound NAD(P)H and fraction of bound FAD with the previous formulas, we are not taking in account the different quantum yield of free and bound forms of the metabolites.

**SUPPLEMENTARY FIGURES**


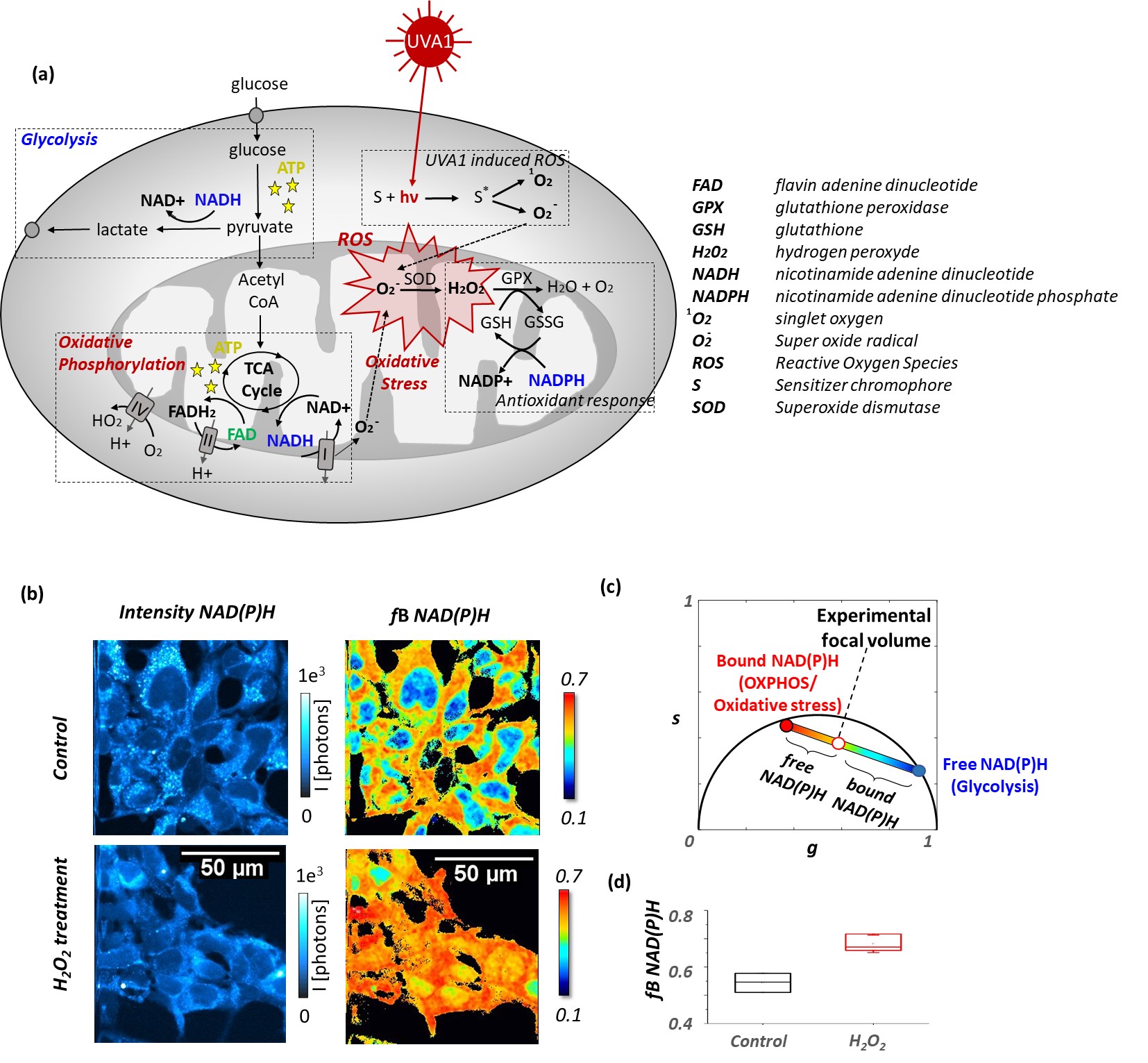


***Figure S1:* Cellular metabolic pathways and Fluorescence Lifetime metabolic trajectory for oxidative stress** (a) Schematic representation of the metabolic pathways inside the cells: Glycolysis, Oxidative phosphorylation (OXPHOS), Oxidative stress and antioxidant response. Glucose breakdown through glycolysis and the TCA (tricarboxylic acid) cycle generates reduced NADH and FADH_2_. Cells with a glycolytic phenotype generate ATP (adenosine triphosphate, yellow stars) rapidly in the cytoplasm and are characterized by a high ratio of NADH/NAD^+^ and a low fraction of bound NADH. Cells relying on Oxidative Phosphorylation convert glucose to pyruvate, which is then oxidized in the TCA cycle generating the majority of ATP in the mitochondria and are characterized by a low ratio of NADH/NAD^+^ and a high fraction of bound NADH. UVA photons with hν energy interact with sensitizer chromophores (S) in the cell, both in mitochondria and cytoplasm, and form reactive oxygen species (ROS) inducing oxidative stress. The absorption of the photon brings the sensitizer from the electronic ground state (S) to a photoexcited triplet state (S*) that directly interacts with oxygen either by energy transfer generating singlet oxygen ^1^O_2_ or by electron transfer generating superoxide radical anion $O_{2}^{-}$. The neutralization of superoxide begins with its rapid conversion into hydrogen peroxide (H_2_O_2_) by superoxide dismutase (SOD). NADPH pool maintains the antioxidant defense by serving as electron donor to glutathione (GSH) to reduce the hydrogen peroxide to water. The principal photosensitizer chromophores (S) in the cells are porphyrins, pheomelanin, flavins and tryptophan. **(b)** Intensity images and fraction of bound NAD(P)H maps of HEK cell in culture treated with 4 mM hydrogen peroxide (H_2_O_2_). Oxidative stress treatment leads to a decrease in NAD(P)H intensity and an increase of bound NAD(P)H in the cells, which is correlated to an increase of NAD(P)+/NAD(P)H ratio. **(c)** Metabolic trajectory between free NAD(P)H (glycolytic phenotype) and bound NAD(P)H (OXPHOS/oxidative stress phenotype) as bound/free NAD(P)H ratio reflects the cellular redox NAD(P)+/ NAD(P)H ratio [[16](#_ENREF_16)]. **(d)** Increase of fraction of bound NAD(P)H in cells treated with hydrogen peroxide (H_2_O_2_). * indicates distributions that are statistically different (**p*<0,05 and ***p*<0,01).

***Figure S2: Histology characterization of UVA1 effects on epidermal and dermal layers of reconstructed human skin***. Representative images of HES (hematoxylin eosin saffron) stained histological sections of reconstructed human skin samples fixed at different time points (30 min, 2 hours and 2 days) after UVA1 exposure. **(a)** Representative HES images at 30 min and 2 hours after 40 J/cm² UVA1 exposure showing no differences in epidermis or dermis layers with respect to the control-sham irradiated samples. **(b)** Representative HES images at 2 days after UVA1 exposure (same samples studied in FLIM imaging experiments) showing the global dose-dependent changes in epidermal and dermal morphology and fibroblast number between control-sham exposed samples and samples exposed to UVA1 at 25 J/cm² and 40 J/cm². **(c)** Representative HES images showing the global changes in epidermal and dermal morphology and fibroblast number between control-sham exposed samples and samples exposed to respectively UVA1 at 40 J/cm² and UVA1 40 J/cm² rays filtered by a sunscreen formulation (same samples studied in FLIM imaging experiments).


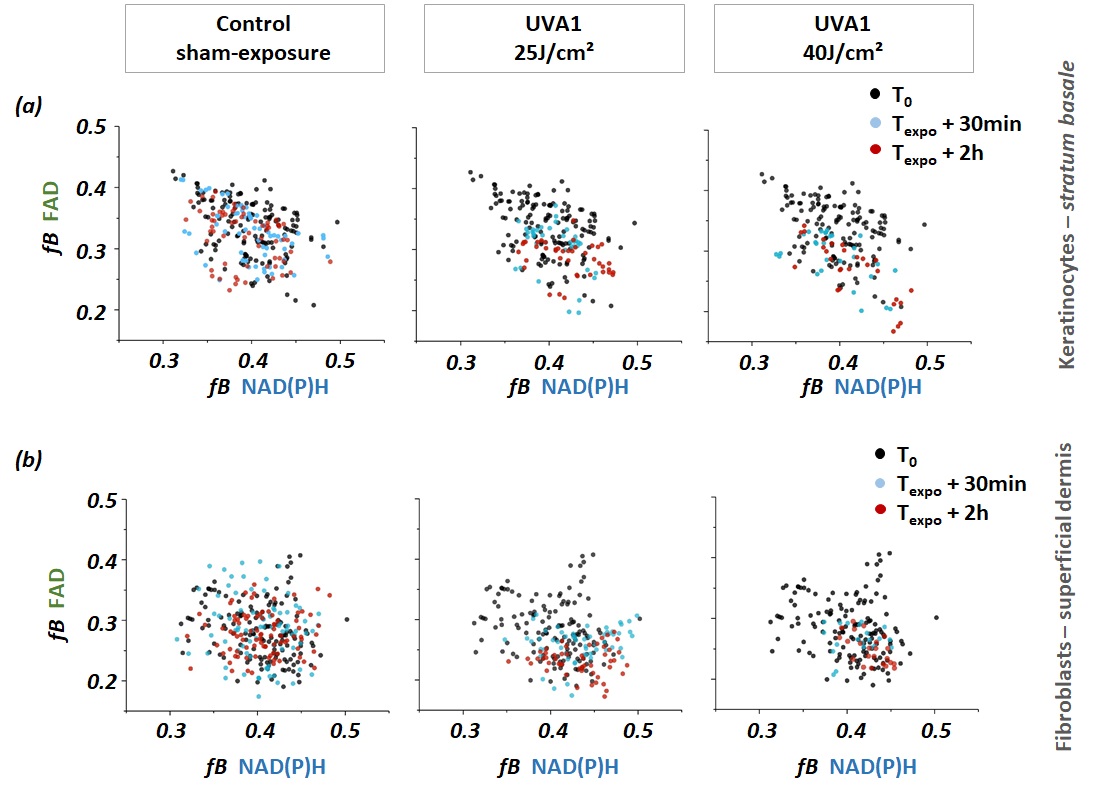


***Figure S3: Single measurements of fractions of bound NAD(P)H (fB NAD(P)H) and FAD (fB FAD) in keratinocytes and fibroblasts upon different UVA1 doses exposure****.* Scatters of fractions of bound FAD vs bound NAD(P)H in **(a)** basal keratinocytes and **(b)** superficial dermis fibroblasts of reconstructed human skin exposed to different UVA1 doses or control-sham exposure. For the measurements in keratinocytes within *stratum basale*, every point represents the average value in a ROIs (dozens of keratinocytes within the acquired image). The corresponding mean ± s.e.m. values are given in Figure 5a and Table 1. For the measurements in fibroblasts within the superficial dermis, every point represents the average value in a single fibroblast. The corresponding mean ± s.e.m. values are represented in Figure 5b and Table 2.


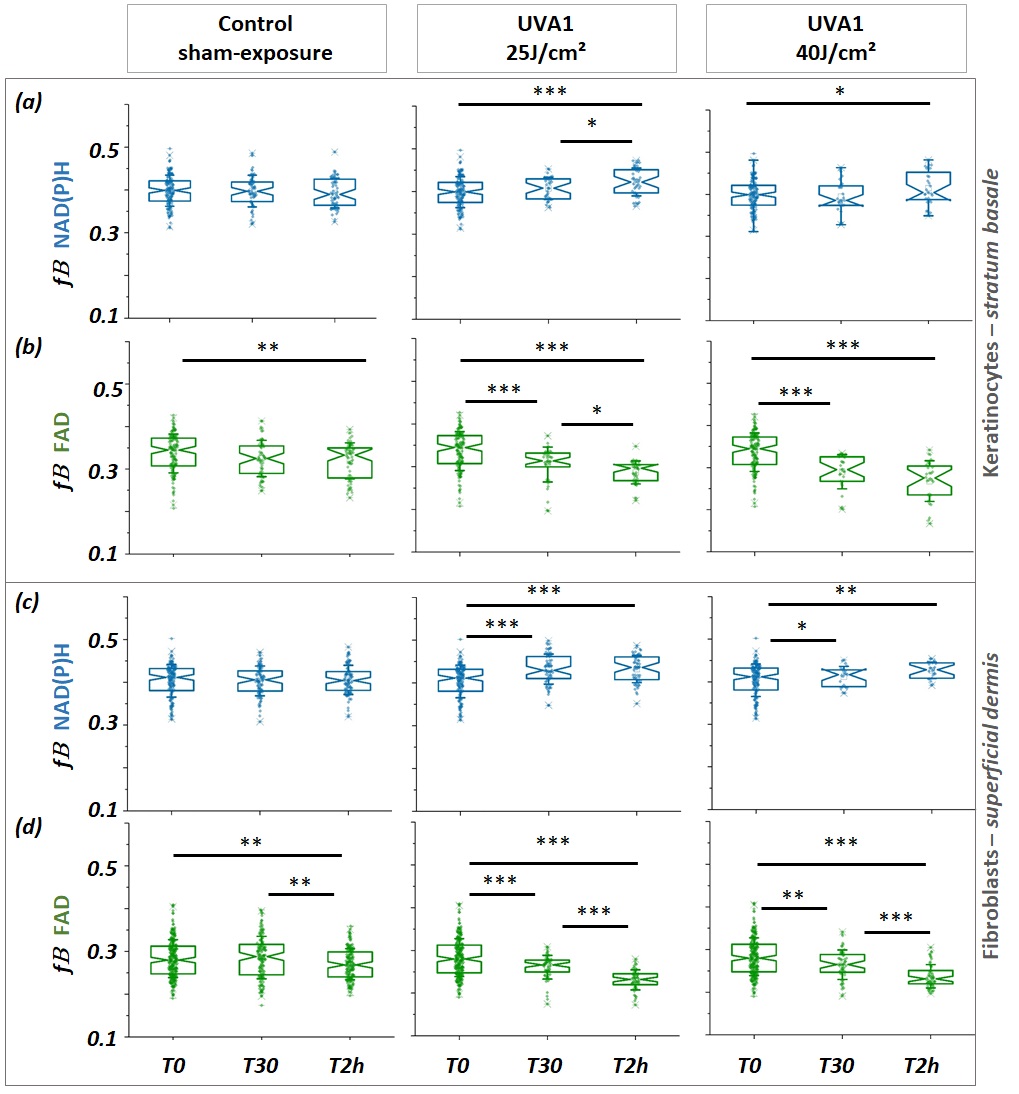


***Figure S4: Changes of fractions of bound NAD(P)H and FAD in basal keratinocytes (a-b) and superficial dermal fibroblasts (c-d) exposed to different UVA1 doses or control-sham exposure***. Data are presented as box plots. * indicates distributions that are statistically different (**p*<0,05, ***p*<0,01 and ****p*<0,001).


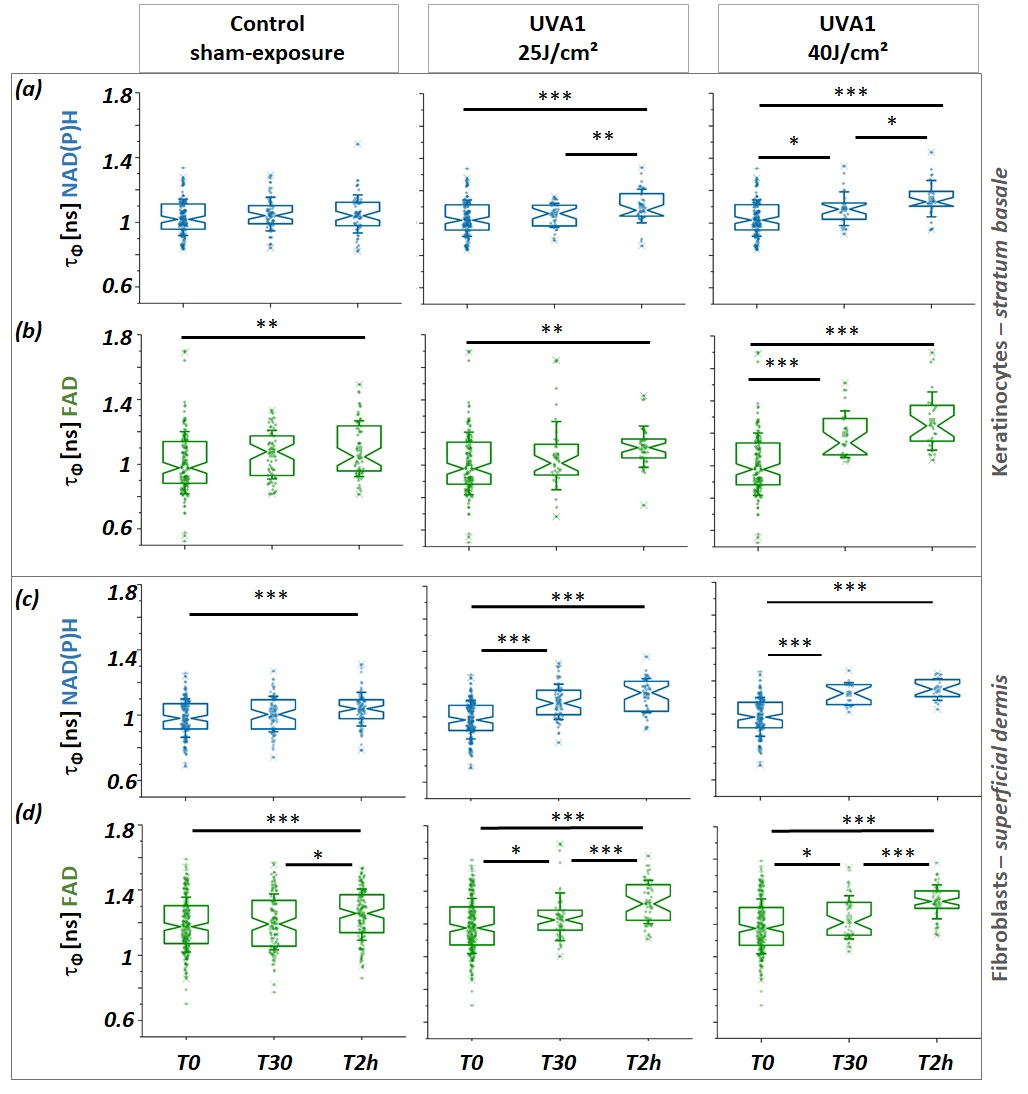


***Figure S5: Changes of NAD(P)H and FAD fluorescence lifetimes (****τ****_Φ_) in basal keratinocytes (a-b) and superficial dermal fibroblasts (c-d) exposed to different UVA1 doses or control-sham exposure***. Data are presented as box plots. * indicates distributions that are statistically different (**p*<0,05, ***p*<0,01 and ****p*<0,001).


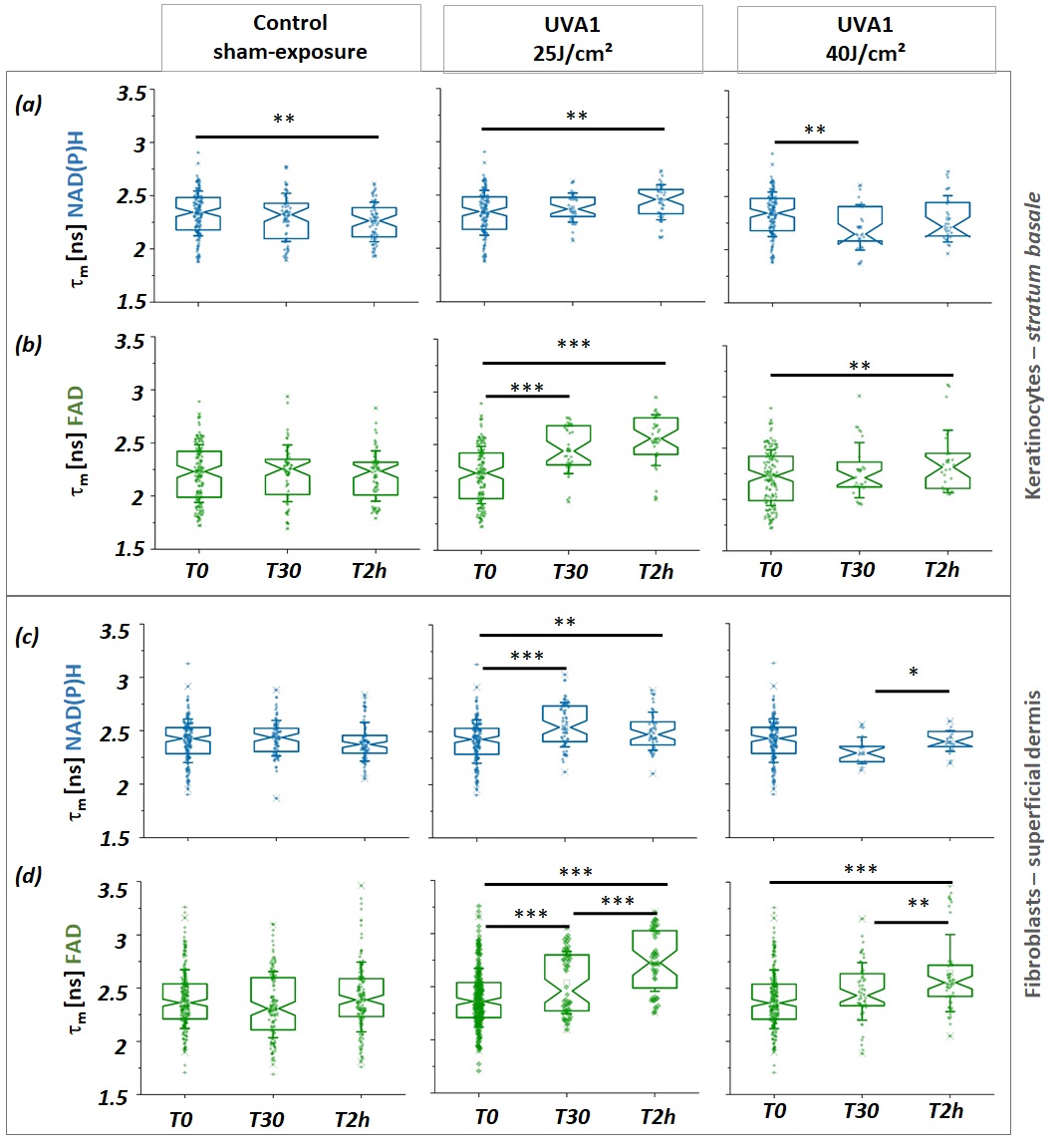


***Figure S6: Changes of NAD(P)H and FAD fluorescence lifetime (τ_m_) in basal keratinocytes (a-b) and superficial dermal fibroblasts (c-d) exposed to different UVA1 doses or control-sham exposure.*** Data are presented as box plots. * indicates distributions that are statistically different (**p*<0,05, ***p*<0,01 and ****p*<0,001).


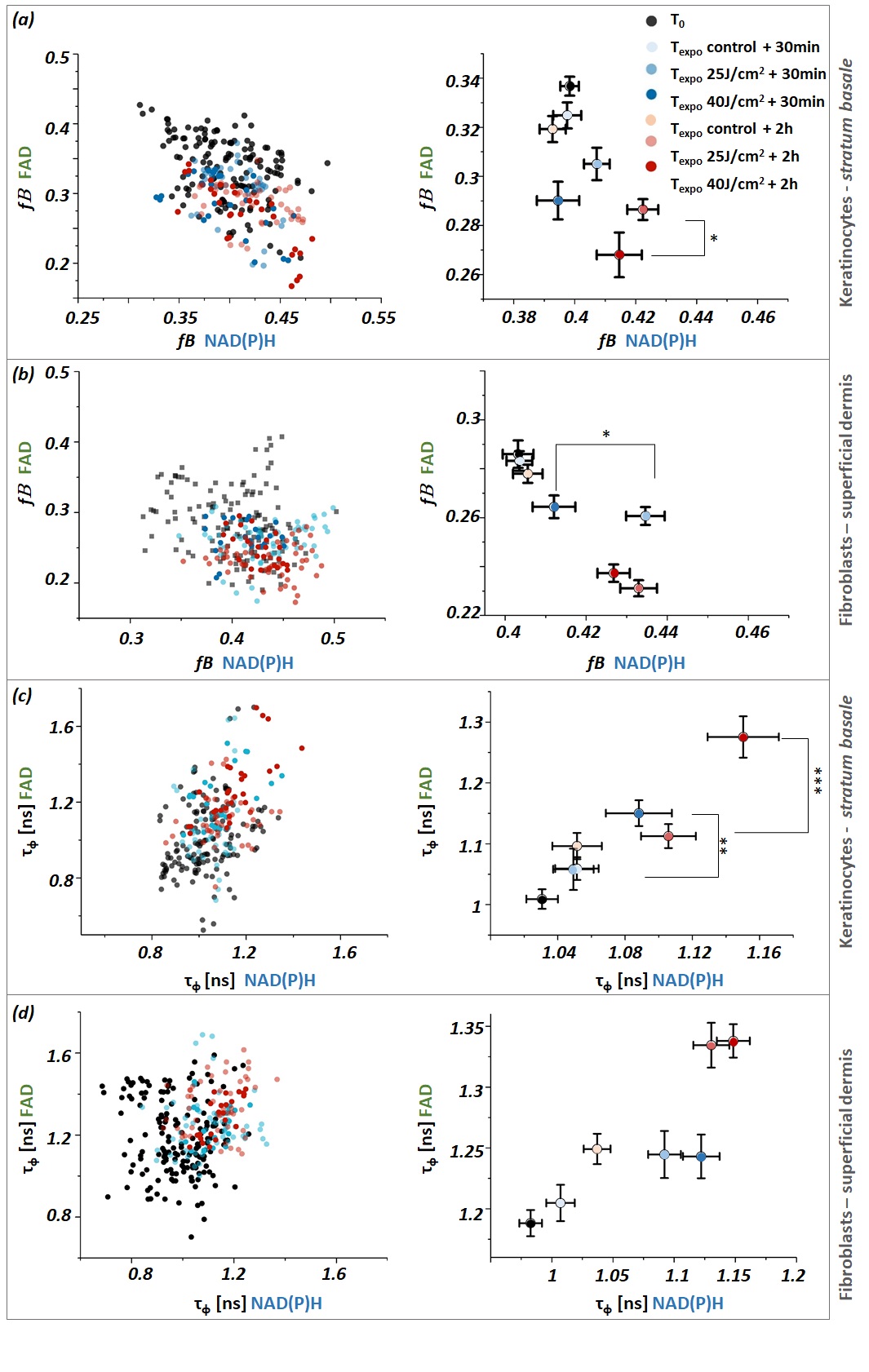


***Figure S7: Single measurements and changes of fB fractions of bound NAD(P)H and FAD and fluorescence lifetimes (τ_Φ_) of NAD(P)H and FAD in keratinocytes and fibroblasts exposed to different UVA1 doses or control-sham exposure***. **(a-c)** Measurements in keratinocytes within *stratum basale*. Every point represents the average value in a ROIs (dozens of keratinocytes within the acquired image). The corresponding mean ± s.e.m. values are represented in the right column. **(b-d)** Measurements in fibroblasts within the superficial dermal layer. Every point represents the average value in a single fibroblast. The corresponding mean ± s.e.m. values are represented in in the right column


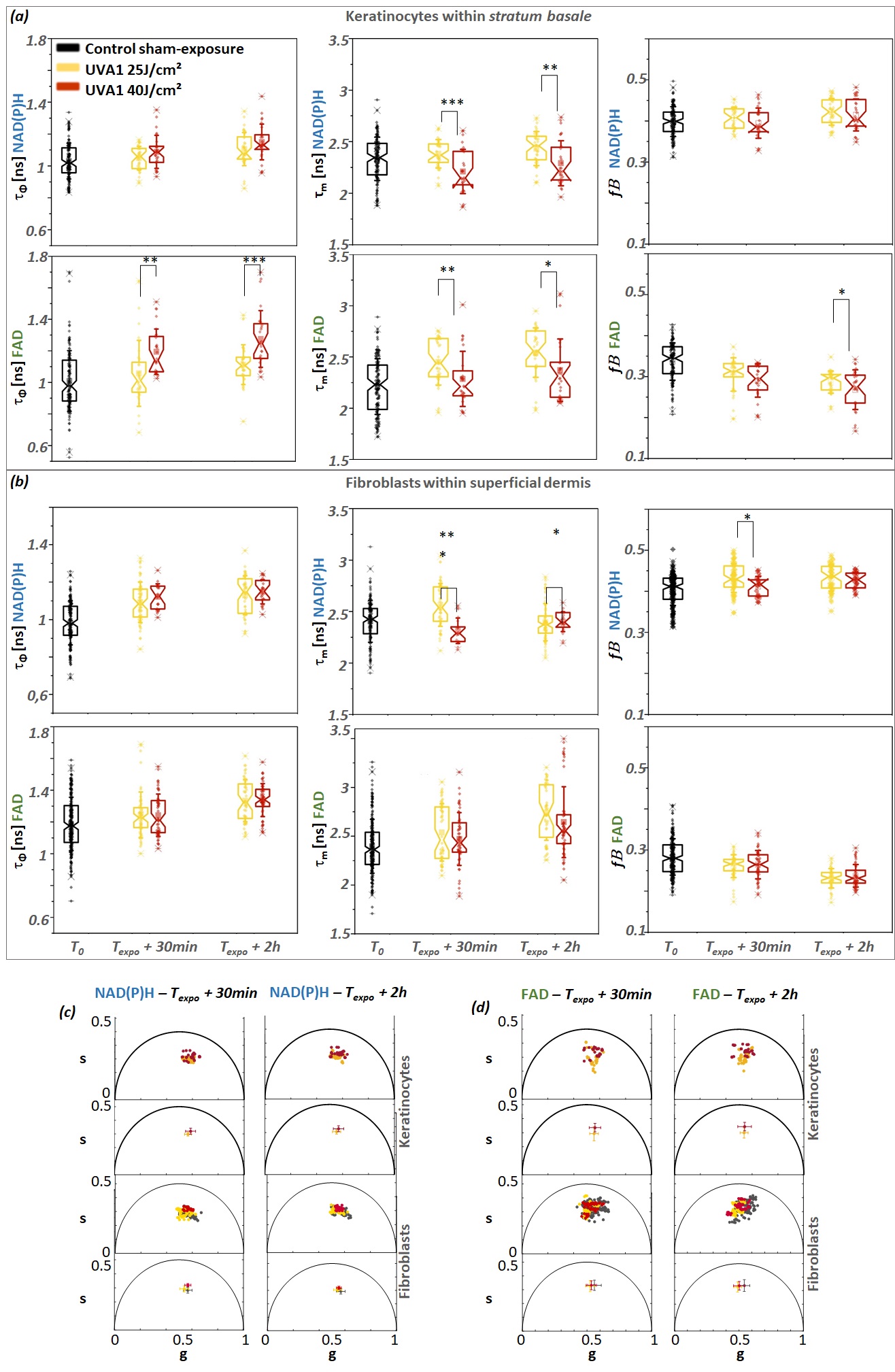


***Figure S8: Sensitivity to UVA1 dose of the NAD(P)H and FAD biomarkers in basal and dermal layers of reconstructed human skin****.* Fluorescence phase τ_Φ_ and modulation τ_m_ lifetimes of NAD(P)H and FAD biomarkers and fractions of bound NAD(P)H and FAD quantification in the basal keratinocytes **(a)** and superficial dermal fibroblasts **(b)** exposed to different UVA1 doses or control-sham exposure. Data are presented as boxplots. * indicates distributions that are statistically different (**p*<0.05, ***p*<0.01 and ****p*<0.001).


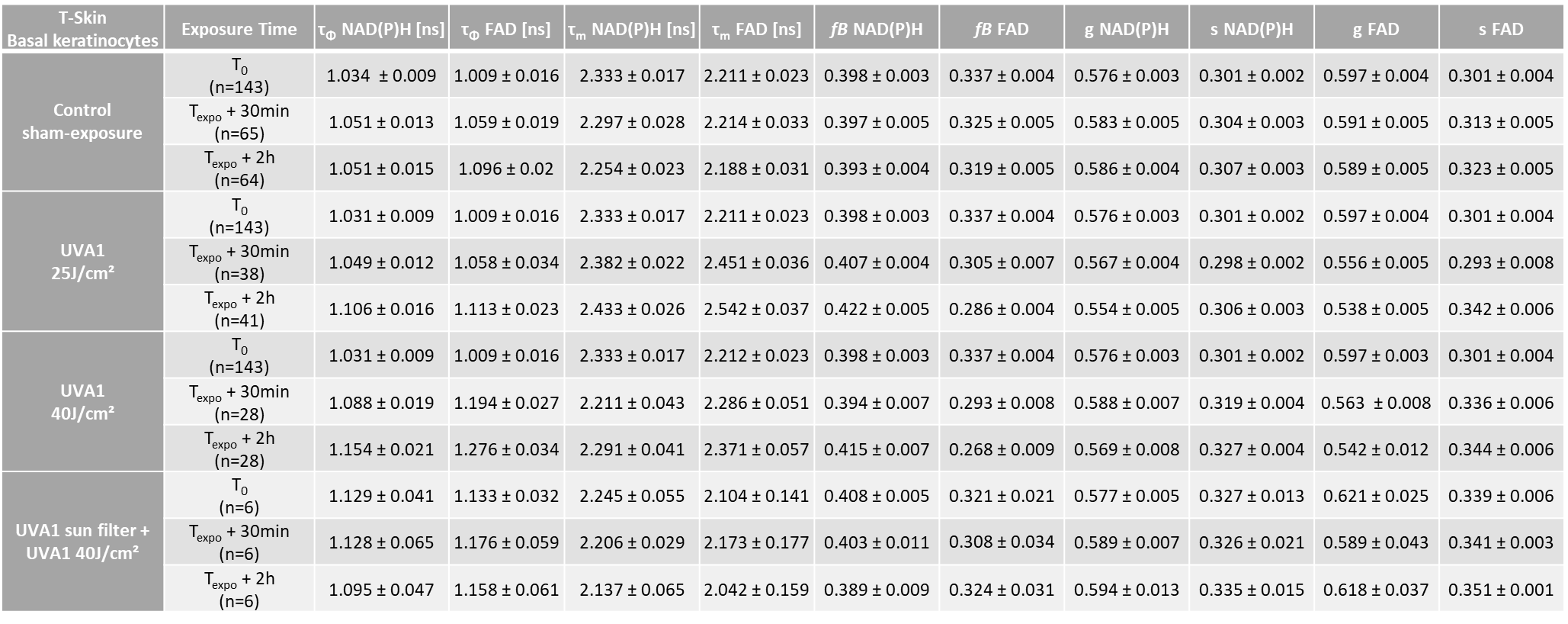


***Supplementary table 1: Data table of FLIM phasor parameters of basal keratinocytes exposed to different UVA1 doses or control-sham exposure***. Data are presented as mean ± s.e.m.


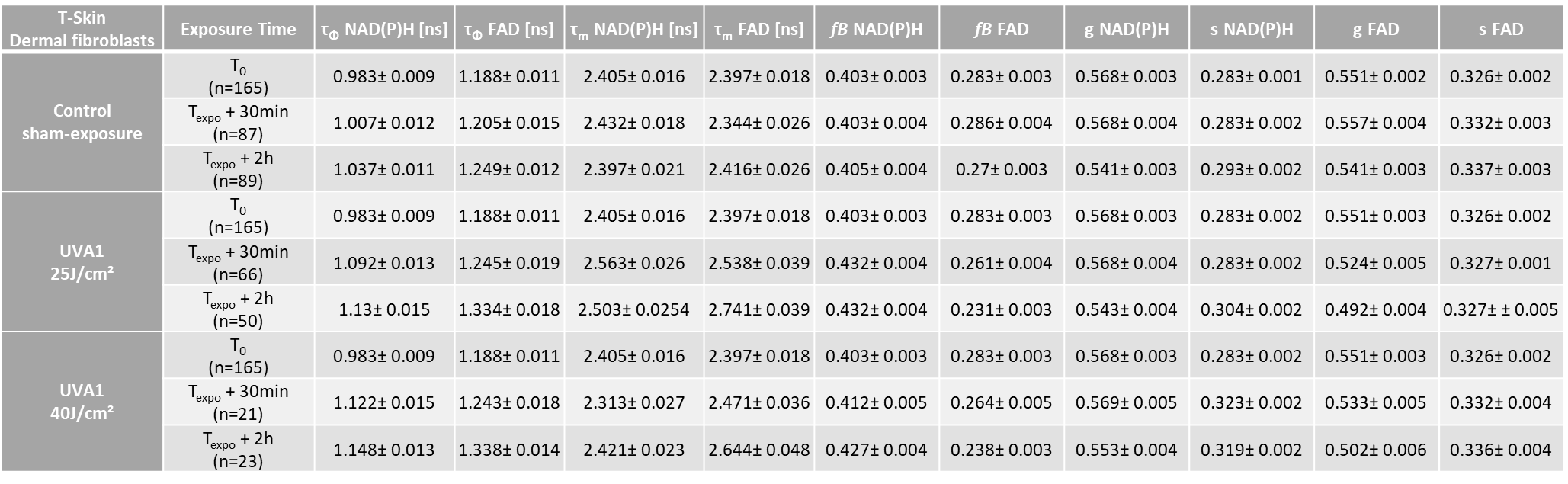


***Supplementary table 2: Data table of FLIM phasor parameters of the superficial dermal fibroblasts exposed to different UVA1 doses or control-sham exposure***. Data are presented as mean ± s.e.m.
